# The roles of apoptosis, autophagy and unfolded protein response in arbovirus, influenza virus, and HIV infections

**DOI:** 10.1080/21505594.2019.1605803

**Published:** 2019-04-30

**Authors:** Parvaneh Mehrbod, Sudharsana R. Ande, Javad Alizadeh, Shahrzad Rahimizadeh, Aryana Shariati, Hadis Malek, Mohammad Hashemi, Kathleen K. M. Glover, Affan A. Sher, Kevin M. Coombs, Saeid Ghavami

**Affiliations:** a Influenza and Respiratory Viruses Department, Past eur Institute of IRAN, Tehran, Iran; b Department of Internal Medicine, University of Manitoba, Winnipeg, MB, Canada; c Department of Human Anatomy & Cell Science, Max Rady College of Medicine, Rady Faculty of Health Sciences, University of Manitoba, Winnipeg, MB, Canada; d Children‘s Hospital Research Institute of Manitoba, Winnipeg, MB, Canada; e Research Institute of Oncology and Hematology, CancerCare Manitoba, University of Manitoba, Winnipeg, Canada; f Department of Medical Microbiology, Assiniboine Community College, School of Health and Human Services and Continuing Education, Winnipeg, MB, Canada; g Department of Biology, Islamic Azad University, Mashhad, Iran; h Department of Clinical Biochemistry, Zahedan University of Medical Sciences, Zahedan, Iran; i Department of Medical Microbiology and Infectious Diseases, University of Manitoba, Winnipeg, MB, Canada; j Manitoba Centre for Proteomics and Systems Biology, University of Manitoba, Winnipeg, MB, Canada; k Health Policy Research Centre, Shiraz Medical University of Medical Science, Shiraz, Iran

**Keywords:** Cell stress, virus infection host response, Bcl2 family protein, caspase

## Abstract

Virus infection induces different cellular responses in infected cells. These include cellular stress responses like autophagy and unfolded protein response (UPR). Both autophagy and UPR are connected to programed cell death I (apoptosis) in chronic stress conditions to regulate cellular homeostasis via Bcl2 family proteins, CHOP and Beclin-1. In this review article we first briefly discuss arboviruses, influenza virus, and HIV and then describe the concepts of apoptosis, autophagy, and UPR. Finally, we focus upon how apoptosis, autophagy, and UPR are involved in the regulation of cellular responses to arboviruses, influenza virus and HIV infections.

**Abbreviation:** AIDS: Acquired Immunodeficiency Syndrome; ATF6: Activating Transcription Factor 6; ATG6: Autophagy-specific Gene 6; BAG3: BCL Associated Athanogene 3; Bak: BCL-2-Anatagonist/Killer1; Bax; BCL-2: Associated X protein; Bcl-2: B cell Lymphoma 2x; BiP: Chaperon immunoglobulin heavy chain binding Protein; CARD: Caspase Recruitment Domain; cART: combination Antiretroviral Therapy; CCR5: C-C Chemokine Receptor type 5; CD4: Cluster of Differentiation 4; CHOP: C/EBP homologous protein; CXCR4: C-X-C Chemokine Receptor Type 4; Cyto c: Cytochrome C; DCs: Dendritic Cells; EDEM1: ER-degradation enhancing-a-mannosidase-like protein 1; ENV: Envelope; ER: Endoplasmic Reticulum; FasR: Fas Receptor;G2: Gap 2; G2/M: Gap2/Mitosis; GFAP: Glial Fibrillary Acidic Protein; GP120: Glycoprotein120; GP41: Glycoprotein41; HAND: HIV Associated Neurodegenerative Disease; HEK: Human Embryonic Kidney; HeLa: Human Cervical Epithelial Carcinoma; HIV: Human Immunodeficiency Virus; IPS-1: IFN-β promoter stimulator 1; IRE-1: Inositol Requiring Enzyme 1; IRGM: Immunity Related GTPase Family M protein; LAMP2A: Lysosome Associated Membrane Protein 2A; LC3: Microtubule Associated Light Chain 3; MDA5: Melanoma Differentiation Associated gene 5; MEF: Mouse Embryonic Fibroblast; MMP: Mitochondrial Membrane Permeabilization; Nef: Negative Regulatory Factor; OASIS: Old Astrocyte Specifically Induced Substrate; PAMP: Pathogen-Associated Molecular Pattern; PERK: Pancreatic Endoplasmic Reticulum Kinase; PRR: Pattern Recognition Receptor; Puma: P53 Upregulated Modulator of Apoptosis; RIG-I: Retinoic acid-Inducible Gene-I; Tat: Transactivator Protein of HIV; TLR: Toll-like receptor; ULK1: Unc51 Like Autophagy Activating Kinase 1; UPR: Unfolded Protein Response; Vpr: Viral Protein Regulatory; XBP1: X-Box Binding Protein 1

## Viruses – general overview

Viruses are the smallest living organisms and all are obligate intracellular parasites that use host cell machinery for their replication. Based on the nature of the virus’ genetic material [whether RNA or DNA], polarity of genome [whether plus-sense (+) or negative-sense (-)] and strandedness [whether single-stranded (ss), double-stranded (ds), or partially ds], viruses are classified into seven groups (Classes 1–7)[1], [2]. Viruses also can be either enveloped or naked, based on the presence or absence of a membrane envelope. Surrounding the genetic material is a protein coat known as the capsid that protects the viral genome against degradative enzymes. Capsids are composed of capsomeres which are arranged such that most capsids are icosahedral (essentially spherical) or helical in shape. As of 2016, the International Committee on Taxonomy of Viruses has reported > 4400 identified viruses in 122 families [3]. Most viruses are extremely specific with regards to the types of host they infect. For example, most plant viruses cannot infect humans or bacteria, and most bacterial viruses (bacteriophages; phages) cannot infect plants or animals. Similarly, with the exception of a few viruses, such as arboviruses (discussed below), most viruses that infect vertebrate animals cannot infect invertebrate insects, and vice versa [4].

Globally, lower respiratory tract viral infections are among the top 10 causes of death, along with cancers, stroke, and diabetes [5]. However, some viruses have beneficial aspects. For example, Reovirus, a Class III dsRNA virus, has oncolytic properties against different types of malignant tumors; thus, it is being explored as a potential tool for cancer treatment [6]. Vaccines are a common defense against virus infections. Vaccines have been derived from whole or subunits of viruses. For example, the hepatitis B vaccine was developed from the viral surface antigens from the inactivated plasma of a carrier [7]. Phage therapy is now the focus of some research groups as an alternative form of treatment of multidrug-resistant bacteria [8]. Baculoviruses, which are insect viruses, infect and kill the larval stage of insects in the order *Lepidoptera*, which are the second most diverse insect Order [9,10]. The caterpillar stages of these insects cause great economic harm. The use of Baculoviruses serves as a natural pesticide. The advantage of using these viruses is that they are insect-specific; thus, they do not harm humans. In addition, unlike chemical pesticides, Baculovirus use does not lead to environmental pollution.

## Example of viruses

The following three virus groups (arboviruses, influenza virus, and human immunodeficiency virus – HIV) will be discussed herein because they represent diverse viruses that employ significantly different molecular mechanisms during their replication, and all are of enormous significance to human health.

**Arboviruses**: These viruses are so named because they are transmitted from animal to animal, or from plant to plant, by invertebrate arthropods (hence arthropod borne). The major insect vectors are primarily found in the tropics and sub-tropical regions; hence approximately one-half of the global population is at risk for these viruses, and the numbers are expected to increase with climate change. The five major families of arboviruses are: *Flaviviridae, Togaviridae, Bunyaviridae, Reoviridae* and *Rhabdoviridae* [11]. Arboviruses are diverse; they have either a plus-sense (*Flaviviridae, Togaviridae*) or negative-sense (*Bunyaviridae* and *Rhabdoviridae*) RNA genome which can either be ss (*Bunyaviridae, Flavividae, Rhabdoviridae, Togaviridae*) or ds (*Reoviridae*). Most of them, except for Reoviruses, have an envelope which is the lipid bilayer acquired while budding out of the host cell. The genome itself of the (+) sense RNA arboviruses serves as the mRNA and is directly translated into a polyprotein which is cleaved by viral and host enzymes into structural and nonstructural proteins. The genomes of (-) sense ssRNA viruses, and the negative-sense strand of the Reovirus dsRNA, must be converted into (+) sense mRNA by the viral-encoded RNA-dependent RNA polymerase (RdRp) before protein translation.

Among arboviruses, 390 million Dengue (DEN) infections are reported globally each year, of which 96 million (67–136 million) manifest as severe clinical forms of the disease [12]. In 2015, Zika virus (ZIKV) was reported to cause microcephaly and microencephaly in infants born to infected mothers [13]. Vaccine development among RNA viruses is generally a challenge because of high mutation levels and lack of proofreading by the RdRp. Thus, compounds with antiviral properties are being screened to determine their antiviral efficacy [14]. Serologic diagnosis for most of these viruses, especially those that are members of the family *Flaviviridae*, is not reliable due to cross-reaction of antibodies produced during infection. Thus, RNA nucleic acid testing by PCR is done to confirm infection, which means countries as well as regions that do not have access to this technology, will have cases which will go unreported, thereby giving a false prevalence rate. In addition, the symptoms elicited in patients who are infected by viruses from the same family, such as DENV and ZIKV, may be very similar, which affects proper diagnosis and clinical administration [15].

**Influenza virus**: Influenza is a (-) sense ssRNA virus in the family *Orthomyxoviridae*. Its genome is segmented, which has important implications in virus evolution and immune escape (discussed below). There are four Classes of influenza; A, B, C and D, and additional genera [16]. However, most human outbreaks are caused by influenza type A virus (IAV). IAV has several subtypes which are based on the two main surface proteins hemagglutinin (H, HA) and neuraminidase (N, NA). There are currently 18 HA types (H1 to H18) and 11 NA types (N1 to N11) [16–18].

The natural reservoir of IAV is waterfowl which generally remain asymptomatic. However, crossover to other animals may lead to the emergence of pathogenic subtypes, such as the 2009 swine flu (H1N1) and the 1968 H3N2 that caused severe forms of the disease. IAV causes both upper and lower respiratory tract infections. Other studies have reported IAV attacking other organs such as the heart and CNS [19,20]. HA facilitates attachment and viral genome is uncoated and transported to the cell nucleus where genome segments are transcribed by viral RdRp into plus-sense mRNA and translated into the various structural and nonstructural proteins needed for IAV replication. New IAV virions bud out of the cell by the help of NA, which cleaves sialic acid from mucins and cell surfaces.

The IAV mode of transmission is mainly by respiratory droplets. High incidences of resistance that develop after prolonged usage make antiviral therapy ineffective. Vaccination is, therefore, the most effective way to prevent IAV outbreaks. Unfortunately, high incidences of mutation in both the HA and NA make complete protection difficult. Mutations arise due to two main genetic phenomena; genetic drift, a result of point mutations due to lack of proofreading by viral RdRp, which usually leads to seasonal differences and epidemics, and genetic shift, which arises because of genetic reassortment between genome segments, which leads to the formation of a totally new virus and often leads to pandemics. Reports by the World Health Organization indicate that between 290,000 and 650,000 respiratory deaths occur annually from seasonal IAV [21]. Currently, a universal flu vaccine that will be effective against any new subtype is the focus of flu researchers and hopefully, if achieved, will end yearly seasonal flu outbreaks [22].

**HIV**: Human immunodeficiency virus (HIV) belongs to the family *Retroviridae*. HIV1 and HIV2 are the two main subtypes; however, most outbreaks involve HIV1. HIV has a diploid (two copies) (+) ssRNA genome. HIV is an enveloped virus, which has two main glycoproteins, GP120, and GP41 which are needed for cell attachment and entry. CD4 + T lymphocytes are the primary targets of this virus. During viral entry, GP120 recognizes and binds to the CD4 molecule, while GP41 recognizes and binds to the cellular co-receptors. The two most common co-receptors are CCR5 and CXCR4. Binding of GP120 to CD4 results in a conformational change in GP41, leading to its binding to co-receptor, followed by cell fusion [23]. Cell fusion results in the release of viral nucleic acid into the cytoplasm which is immediately reverse transcribed into dsDNA by viral-encoded Reverse Transcriptase (RT). Newly synthesized viral dsDNA moves into the host cell nucleus where viral-encoded Integrase integrates it into the host DNA. This molecule, now called provirus, uses a host RNA polymerase to produce mRNA, which is translated into viral proteins. Many viral proteins are produced as polyproteins that are cleaved into mature viral proteins by viral-encoded Protease and together, with viral progeny genetic material, bud out of the cell, acquiring an envelope from the host cell with both GP120 and GP41, and some host proteins, embedded in the viral membrane.

The acute stage of HIV infection involves increases in viral load which leads to depletion of the CD4 + T lymphocytes. This stage lasts for approximately 10 weeks from the day of infection. Seroconversion occurs at this stage and transmission is very high. The virus then moves from the blood into lymphoid organs. This stage is known as clinical latency. There is a high level of replication with approximately 10 billion viral copies produced each day. This stage can last for 10 years or more, depending on the immune status of the patient. CD4 T cell numbers may increase at this stage. The late stage involves a sudden increase in viral load with a drastic decrease in CD4 + T cell counts leading to AIDS (acquired immunodeficiency syndrome) which is characterized by opportunistic infections and malignancies.

The major challenge in HIV treatment and management is the continuous survival of latently infected cells which indicate incomplete removal of infectious viral particles from an infected individual. HIV is the fourth leading cause of death in developing countries [5] and so far has claimed more than 35 million lives globally [24]. In 2017, 940,000 deaths because of HIV infection were recorded [24]. No effective vaccine has been developed due to high levels of mutation within the viral genome [25]. Thus, antiviral therapy, involving various inhibitors against fusion, Integrase, and Protease (combination therapy), are used for management of infected patients. Some efforts are in early stages and involve the use of combination antiretroviral therapy (cART). A recent study showed the impact of cART in HIV-1 infection by examining the JAK-STAT signaling pathway which plays a crucial role in host innate immunity against HIV-1 [26]. The JAK-STAT signaling pathway is involved in Toll-like receptor (TLR)-mediated pathogen recognition, IRF activation and the induction of IFN-inducible genes such as MxA and ISG56. Liu and colleagues noticed that HIV-1-infected subjects had lower levels of the TLRs, IRFs and the cellular anti-HIV factors. Therefore, they postulated that deficiencies in the JAK-STAT pathway may play a role in the immunopathogenesis of HIV-1 disease. These new directions are focused on latency-reversing agents to activate immunotherapies, gene therapies, and therapeutic vaccines to eliminate persistent HIV reservoirs or induce effective immune control of HIV infection [26].

## Cell stress response to viral infections

Virus-induced stress responses are the major focus of this review. Cell stress responses include a wide range of cellular mechanisms intended to either reduce cellular stress-induced damage, or if the damage is beyond repair, direct the cell towards cell death. Cellular stress responses can be protective in nature (e.g. heat shock response or unfolded protein response (UPR)), or they may be pro-cell death, such as programmed cell death, or necrosis [27]. Autophagy is another cellular stress response pathway. Autophagy is a multistep process that involves degradation and recycling of proteins and organelles trapped in autophagosomes targeted to fuse with lysosomes. Autophagy helps cells adapt to stress by either preventing cell death or promoting it (autophagic cell death) [27].

Since viruses manipulate cellular machinery for the production of progeny viral particles, they heavily depend on cellular proteins like heat shock proteins for proper folding and assembly of the viral proteins [27,28]. Viruses like cytomegalovirus (CMV – a herpesvirus) and Flaviviruses (DENV, ZIKV and Japanese encephalitis virus (JEV)) induce the host UPR response, which promotes increased protein folding and successful viral replication, while other viruses like herpes simplex virus type 1 (HSV-1) work to downregulate the IRE1/XBP-1 branch of the UPR pathway [29,30]. These stress-induced responses are crucial in the manifestation of virus-induced effects like ZIKV-induced development of microcephaly, influenza virus-induced inflammatory damage, and reactivation of HSV-1 in previously quiescent human fibroblasts [31–34]. Modulation of autophagic flux, as well as induction of intracellular oxidative stress, are also used by viruses like ZIKV and influenza virus to promote viral replication and increase virally induced cell damage [31,35].

Here we first provide a brief introduction to cellular stress responses, including apoptosis, autophagy and unfolded protein response and then we discuss how these mechanisms are involved in the regulation of viral infection in arboviruses, influenza virus, and HIV.

## Apoptosis

The term apoptosis was introduced by Kerr in 1972, and is based on the Greek word which means ‘falling off or dropping off” an analogy to leaves falling off trees or petals dropping off flowers [36]. Apoptosis is a highly regulated form of cell death in which the cell contains the necessary information to die on its own. Once the decision to die is taken, there is a proper execution of the apoptotic program, which requires coordinated activation and execution of several other multiple subprograms [37]. That is why the apoptotic process is rightly called “programmed cell death”. Apoptosis is characterized by cell shrinkage, followed by chromatin condensation, nuclear fragmentation and plasma membrane blebbing. There are two commonly described apoptotic pathways; these are extrinsic apoptosis and intrinsic apoptosis [38].

### Extrinsic apoptotic pathway

The extrinsic apoptotic pathway is initiated by the activation of death receptors present on the cell surface. These death receptors belong to the subset of tumor necrosis factor receptor (TNFR) superfamily. There are six well-known death receptors that can regulate apoptosis either directly or indirectly. These include TNFR1, Fast/Apo/CD95 (TNFRSF6), DR3 (TNFRSF25), DR4 (TNFRSF10A), DR5 (TNFRSF10B) and DR6 (TNFRSF21) [39,40]. Upon stimulation by the binding of the corresponding ligand, these death receptors can ligate and activate apoptotic signals. Initially, they trigger the apoptotic cascade by forming a death-inducing signaling complex (DISC) which often contains the adaptor protein FADD and the apoptosis initiating protease caspase-8. Once caspase-8 gets activated, it, in turn, activates other executioner caspases such as caspase-3 and −7 and these activations eventually commit the cells to undergo apoptosis [40].

### Intrinsic apoptotic pathway

The intrinsic apoptotic pathway is tightly regulated by the Bcl-2 gene family of proteins. These proteins control the release of specific caspase activating factors from the mitochondria. The Bcl-2 family is divided into various subsets depending upon Bcl-2 homology (BH) motifs. There are proteins that contain a single BH motif; these include Bid, Bad, Bik, Buff, Bid, PUMA, and NOXA [41,42]. Proteins such as Box, Bok and Back contain three BH domains and Bcl-2 and Bcl-xL proteins contain four BH domains [41]. Some of these Bcl-2 family proteins can act as cell death agonists, such as Bid, Bad, Bax, and Bak, whereas Bcl-2 and Bcl-xL can act as antagonists to cell death [41,43,44]. The intrinsic apoptotic pathway is activated by a variety of intracellular signals including DNA damage due to oncogenic stress. This pathway is associated with the disruption of mitochondrial outer membrane potential (MOMP) and eventually leads to the activation of caspase-9. The caspase-9 activating platform contains APAF1 and cytochrome C. These intracellular signals, in turn, activate Bax and Bak, which help in the formation of pores in the outer mitochondrial membrane which disrupts the mitochondrial membrane potential and causes the release of cytochrome C from the mitochondrial inner membrane. The released cytochrome C forms a complex with APAF1 and leads to the activation of caspase-9. Caspase-9 activation, in turn, activates other executioner caspases eventually leading to apoptotic cell demise [45].

### Significance of apoptosis

Apoptosis (programmed cell death I) plays a crucial role in a wide variety of physiological processes. During fetal development, and in the course of development of an organism into a mature adult, many cells are produced in excess and these will eventually undergo programmed cell death as complete and mature organs and tissues are formed. As a whole, apoptosis plays a crucial role in the development of, and in maintaining the equilibrium of the body [46,47]. Apoptosis also plays an important role in the proper development of the immune system. For example, in the process of T-cell proliferation, only matured cells are positively selected, but immature cells are removed by the process of apoptosis [48,49]. In addition, apoptosis plays a vital role in eliminating dangerous cells such as tumor cells, cells infected with pathogens and cells defective in their function [48,50]. A defect in the apoptosis program, or deregulation of the apoptotic process, results in cancer, autoimmune diseases and spreading of viral infections [38]. Excessive apoptosis could lead to the development or enhancement of neurodegenerative diseases, ischemic diseases (stroke, myocardial infraction) and AIDS [51]. Due to the importance of programmed cell death in various biological processes, this phenomenon has been widely studied in mammals, insects [52], cnidarians [53] and nematodes [54]. It has been shown that even plants [55–57] and monocellular organisms like yeast [58] also can undergo apoptosis.

## Autophagy

The term autophagy is defined as self-eating. It sequesters, degrades and recycles cellular materials. The autophagy function is evolutionarily conserved in yeast, plants, and mammals as a basic stress-response and degradation mechanism. The role of autophagy has been widely investigated in humans, as it plays crucial roles in maintaining optimum functional conditions at the cellular and organismal level [59]. Autophagy is necessary and beneficial for cells because it removes damaged cell organelles, prevents the buildup of toxic protein aggregates and provides the cell and organism with the bioenergetic substrates needed to survive [60]. Thus, autophagy is constitutively activated in normal physiologic cell conditions, although its level is cell type dependent [61]. Autophagy is involved in a variety of physiological processes including cell differentiation and development, starvation and degradation of aberrant structures which ultimately maintains essential cellular homeostasis [62,63]. Physiologic autophagy plays beneficial roles on several basal cellular mechanisms in different organs through its intracellular catabolism activities, while pathological autophagy influences outcomes in disorders such as neurodegeneration, immunity, and cancer [59,64–69]. Historically, research in the autophagy field was initiated by studies which characterized the lysosome; this led to our current knowledge about regulatory and molecular aspects of autophagy [70]. Autophagy can be defined as a catabolic process which degrades and recycles cytosolic materials. It is a highly regulated cellular process divided into three main types: Macroautophagy, Microautophagy and Chaperone-Mediated Autophagy (CMA) [59,71,72].

Macroautophagy involves double-membrane autophagosomes that engulf different cargos like organelles and cytoplasmic proteins. These autophagosomes sequester their cargo to lysosomes where the cargo is degraded [73]. Microautophagy results in the direct engulfment of substrates through lysosomal or endosomal membrane invagination, and the substrates then are degraded by lysosomal proteases [74]. CMA acts in a very selective way and does not use membranes to engulf the cargo. This makes it different from macroautophagy and microautophagy. Proteins targeted by CMA contain a pentapeptide motif containing KFERQ sequence (Lys-Phe-Glu-Arg-Gln) that is detected by cytosolic heat shock cognate 70 kDa protein (hsc70). Hsc70, together with the lysosomal-associated membrane protein 2A (LAMP2A) receptor, helps the cargo be transferred into lysosomes through their membranes [75]. Pathways of selective or nonselective autophagy include glyophagy (glycogen), lipophagy (lipids), pexophagy (peroxisomes), nucleophagy (nucleus), reticulophagy (endoplasmic reticulum), mitophagy (mitochondria), xenophagy (intracellular pathogens) ribophagy (ribosomes) and zymophagy (zymogen granules) [76–80].

### Macroautophagy

Macroautophagy was discovered in the late 1950s using morphological techniques [81]. Being a principle degrading mechanism of the cell, macroautophagy contributes to the survival of cells under stressful conditions [69,82]. Briefly, the cargo is sequestered into autophagosomes followed by their delivery to lysosomes for degradation [15]. There are many proteins involved in the autophagy pathway including ATG proteins. Thirty-two ATG proteins have been identified which play crucial roles in different steps of autophagy activation [81,83–85].

Autophagosome formation includes three steps: initiation, nucleation, and expansion. The first step in formation of autophagosome is at the phagophore assembly site (PAS) (isolation membrane) where proteins of the unc-51 like autophagy activating kinase 1 (ULK1) complex combine in order to begin autophagosome formation [86]. In the next stage (nucleation), activated ULK complex targets a class III PI3K complex to contribute to the production of a PI3K pool that is specific to autophagosomes [87]. In the next step, phosphatidylinositol (PI) 3-phosphate (PI3P)-enriched membrane domains (omegasomes) are formed which then expand to form the double-membrane autophagosome [88]. In the final stage, the autophagosome membrane recruits the ATG12–ATG5–ATG16 complex, which facilitates microtubule-associated protein 1 light chain 3 (MAP1LC3; LC3) lipidation with phosphatidylethanolamine (PE). The isolation membrane expansion is dependent on LC3 (the mammalian homolog of yeast *ATG8)*. Deacetylation of LC3 and cytosolic translocation is essential for its lipidation during starvation-induced autophagy [89].

Almost 30 genes from the autophagy-related (ATG) family regulate the autophagy process. These genes were first identified in yeast and later their orthologues were identified in humans [90]. Upon activation of the autophagy process, a series of Atg protein complexes orchestrate the formation of double membranous vesicles called autophagosomes that capture cytoplasmic cargo. Cargo receptors bind both cargo and to the autophagosome LC3-II component and help in the process of sequestration [91]. Fusion between the autophagosome and lysosome leads to the formation of the autolysosome. Lysosomes contain hydrolases that can help in the degradation of cargo. Cargo is then degraded into amino acids, nucleosides, fatty acids, and sugars and they are released into the cytosol for recycling [91].

The origin of autophagosome membranes in yeast is likely to be *de novo* [92,93]. However, this is a contentious subject in mammalian cells. There are a wide range of sources that can contribute to autophagosome formation (e.g.; ER–Golgi intermediate compartments, ER–mitochondria junctions, mitochondria, endosomes, and the plasma membrane). However, evidence supports the notion that isolation membrane nucleation occurs at a distinct site and emanates from the ER [94]. Formation of the autophagosome can be triggered by different types of cellular stress, such as amino acid starvation, growth factor deprivation and other types of external stressors [81]. During the biogenesis of autophagosomes, either portions of the cytoplasm (bulk autophagy) or distinct cargo molecules (selective types of autophagy) are sequestered in the interior of these transport carriers and enclosed during phagophore formation and expansion [81]. Ultimately, autophagosomes either fuse directly with lysosomes to expose their content to hydrolytic enzymes, or first fuse with endosomes to form intermediate compartments called amphisomes before the autophagosomal cargo reaches the lysosome where cargo is degraded and metabolic molecules are delivered to the cytoplasm [95].

Autophagy plays a prominent role in the selective removal of damaged organelles and unfolded proteins [96]. It was believed that autophagy induced by growth factor deprivation acts in a non-selective manner. However, the currently accepted theory is that autophagy sequesters its cargo (organelles, unwanted proteins, etc.) in a very selective mechanism [97]. Generally, changes in cellular metabolic processes cause non-selective autophagy while alterations in homeostasis (such as damaged mitochondria, misfolded proteins, bacterial or viral infection) trigger selective autophagy [98,99]. During selective autophagy, cargo is attracted through five well-known special receptors [p62 (SQSTM1), NBR1, NDP52, OPTN, and NIX] which recognize the degradation signals on cargo. Most of these receptors have an LC3-interacting region (LIR) [100] and a ubiquitin-binding domain (UBD) [101]. This signal in mammals is usually ubiquitin which binds to the receptor UBD [102]. p62 (SQSTM1) is a cargo receptor which greatly contributes to the removal of protein aggregates; a process called aggrephagy. This process is also dependent on the UBD and LIR2 [100]. Furthermore, organelles are also targets of selective autophagy. As an example, mitophagy is involved in the process of damaged mitochondrial degradation and recycling [98,103]. Recent studies have identified the presence of receptors involved in mitophagy, such as BCL2/adenovirus E1B 19 kDa protein-interacting protein 3 (BNIP3) and ATG32 in mammals and yeast, respectively [104–106]. They regulate mitophagy via phosphorylation of some of their residues, and they use LIR in order to sequester mitochondria [107]. One important concept in the study of autophagy is “autophagic flux”, which is the measurement of the rate of autophagic degradation activity. The rate of the degradation activity is directly related to the respective rates of degradation [108].

A basal level of autophagy acts as an intracellular quality control system in normal conditions by protecting the cell from unwanted and damaged proteins and organelles [109–111]. Autophagy serves as an adaptive and cytoprotective response upon activation by various stimuli such as oxidative, genotoxic and nutritional factors [73,112]. This has been further proved by the observation that cells with non-functional autophagy (chemical or genetic intervention) do not have the necessary ability to adapt to the stressful conditions [110,112]. Therefore, due to its cytoprotective role in the cell, autophagy serves as a defensive mechanism against different abnormalities like tumorigenesis and also against virus infections. Basal autophagy is also vital for the health and homeostasis of other cell types like neurons and muscle cells, as it has been observed that autophagy dysfunction can lead to the formation of inclusion bodies because of damaged protein aggregation and result in the development of neurodegenerative and cardiac disorders [113]. In addition, autophagy contributes to ER homeostasis through a process called reticolophagy, where some areas of the ER and even part of the nucleus, are targeted and sequestered by selective autophagy. Reticolophagy generally occurs under nitrogen-deprived conditions, and ATG39 and ATG40 are required in the reticolophagy process [114]. These observations show how autophagy can be a determinant factor in controlling cellular metabolic systems both in healthy and unhealthy cells [71].

### Microautophagy

Microautophagy is a non-selective process in which the proteins that are required to be degraded are transferred into the lysosome by being bent into its membrane. Microautophagy occurs without the involvement of autophagosomes. This form of autophagy is the least studied of all currently known autophagic processes [90]. As the name indicates, mainly small molecules are the substrates for microautophagy.

### Chaperone-mediated autophagy

In chaperone-mediated autophagy (CMA), a cytoplasmic chaperone mediates lysosomal-associated membrane protein 2A (LAMP 2A)-dependent uptake of unfolded proteins. In CMA, cytosolic proteins with the pentapeptide motif KFERQ are recognized by heat shock cognate protein 70 kDa protein (HSC70), also known as HSPA8 [115,116]. These proteins and HSC70 from the chaperone complex and translocate into the lysosome via LAMP2A. These proteins are degraded in the lysosomes. CMA has been implicated in cancer [115].

## Autophagy and viral infection

Autophagy has harmonized with the immune system to enhance and regulate numerous antiviral immunological responses. Autophagy represents an ancient form of antiviral defense that plays a major role in antiviral defense in systems in which other antiviral mechanisms are absent [117].

Following viral infection, autophagy initially triggers an innate immune response by cooperating with a pattern recognition receptor (PRR) to induce interferon production and dispose of attacking viruses [118]. Then, autophagy coordinates adaptive immunity by delivering antigens derived from the virus for presentation to T lymphocytes [119]. However, during evolution, some viruses have acquired the ability to hijack autophagy, use autophagy-generated metabolites and, in some cases, convert the autophagosome into their “home” during replication. Given that viral infection causes cell stress, autophagy is a frequent by-product of viral infection [120].

The intracellular endosomal TLRs are primary detectors of host defense against viral pathogen-associated molecular patterns (PAMPs). They may recruit different adaptors to activate nuclear factor-κB (NF-κB) and interferon regulatory factors (IRF-3) for IFN production [121]. During viral infection, TLR activation tends to induce autophagy by binding of MYD88 or TRIF to Beclin 1 which disturbs BCL-2 interaction to improve IFN production, whereas the negative regulation of autophagy helps terminate and inhibit TLR signaling by promoting the selective degradation of TRIF [122]. Upon viral infection, the cross-talk between PRRs and autophagy leads to the activation or inactivation of various intracellular signaling pathways, which generates an optimal antiviral environment [120].

Following the presentation of viral antigen fragments on MHC molecules in antigen-presenting cells (APCs), which are then recognized by T cells, the adaptive immune response is initiated. MHC class I molecules present intracellular antigens after they have been processed by the proteasome and transported into the ER by the antigen peptide transporter (TAP). There is an additional mechanism for loading exogenous antigens onto MHC class I molecules in a process known as cross-presentation. Cross-presentation modulates trafficking and processing of phagocytosed antigens from the endosome to MHC I, and autophagy induces antigen packaging in donor cells for release to APCs [120]. By focusing on the mechanisms by which autophagy or *ATG* gene products are utilized by the mammalian immune system to coordinate the antiviral defense, it was found that deletion of the proteins ATG5 and ATG7 decreased mouse survival following intracranial injection with SINV, providing further evidence that autophagy is required in antiviral defense *in vivo* [123].

However, despite all these protective cellular strategies, some viruses have evolved several tactics to escape, inhibit or even recruit multiple steps of the autophagy pathway to their benefit. Several classes of viruses have been reported to manipulate the autophagosomes and induce autophagy during infection, using them as a physical platform for viral replication machinery by recruiting autophagy to protect itself from detection by the host immune system, concentrate essential intermediates and also achieve their maximal viral replication in vitro [117].

Influenza A virus (IAV) antagonizes autophagy induction. Its M2 protein blocks autophagosome-lysosome fusion [124]. Disruption of the M2–LC3 interaction decreases virion budding and stability. NS1, a multifunctional IAV protein, also stimulates autophagy indirectly by increasing the synthesis of HA and M2 [125]. HIV-1 induces autophagosome formation and HIV-1 Gag colocalizes with LC3, but HIV-1 Nef blocks autophagosome-lysosome fusion by interacting with Beclin 1 to sequester transcription factor EB (TFEB) in the cytosol, thus inhibiting autophagosome maturation [126]. These viruses avoid autophagy-dependent degradation and possibly interfere with innate recognition and presentation of viral antigens on MHC molecules [117]. Flaviviruses also use ER-derived membranes for viral replication [120].

There are also multiple viruses that exploit lipophagy. DENV infection increases the number of lipid droplets per cell. These dropleys contain viral capsid proteins, suggesting that they provide a platform for nucleocapsid formation and viral replication [127]. Moreover, DENV infection induces lipophagy, which depletes stored triglycerides and increases β-oxidation and energy production for viral replication [128].

Although the process remains unclear, autophagic membranes enable some viral particles to reach the extracellular space either in exosomes or viral envelopes. Flaviviruses normally use autophagic membranes both for replication and exocytosis through multivesicular bodies (MVBs). DENV reduces p62, an autophagy receptor, via proteasomal degradation, to support its replication [129].

Each virus uses distinctive strategies to simultaneously escape destruction by autophagy, while deriving structural and nutrient benefits provided by autophagy. All further details of specific viruses’ interference with autophagy are described below in the related sections.

## Unfolded protein response pathway

The endoplasmic reticulum (ER) is the intracellular organelle which is important in regulating various metabolic activities such as metabolism of carbohydrates, lipid biogenesis and calcium homeostasis, protein synthesis and post-translational modification of many secretory and membrane proteins. The ER contains a large network of tubules, sacs, and cisternae which extend from the cell membrane through the cytoplasm to the nuclear envelope through a continuously connected network [130–132]. The ER is the main sub-cellular compartment involved in proper folding of proteins and their maturation. About one-third of the total cellular proteins are synthesized in the ER. Many different perturbations can alter the function of the ER leading to unfolding or misfolding of proteins in the ER. This condition is referred to as ER stress [133,134]. The ER creates a series of adaptive mechanisms in order to prevent cell death complications and these together are referred to as unfolded protein response (UPR) [133,135]. UPR involves activation of three major canonical ER stress sensors, namely inositol-requiring enzyme I (IRE1), protein kinase RNA-activated like ER kinase (PERK) and activating transcription factor 6 (ATF6). These ER stress transducers are localized to the ER membrane [131].

***IRE1***: IRE1 is a type I transmembrane protein receptor having a N-terminal ER luminal sensing domain. The cytoplasmic C-terminal region contains both an endoribonuclease domain and a Ser/Thr kinase domain [130,131,136]. There are two homologs of IRE1, namely IRE1α and IRE1β. Activation of IRE1 involves first dissociating itself from Grp78. Then, IRE1 undergoes dimerization, oligomerization, and trans-autophosphorylation, which leads to conformational changes and activation of its RNase domain [133,134]. Activated IRE1 excises a 26-nucleotide intron region from mRNA that encodes the transcription factor X-box binding protein 1 (XBP1). Dissociation of the 26-nucleotide intron region from XBP1 leads to a shift in the coding reading frame and produces a more stable form of XBP1 called XBP1 spliced form (XBP1s) [133,137]. The IRE1-XBP1’s signaling axis modulates pro-survival responses by targeting many genes involved in protein folding, maturation and ER-associated degradation [130,138]. XBP1 also modulates phospholipid synthesis which is required for ER expansion under ER stress [139]. Some examples of XBP1 target genes include ERdj4, P58^IPK,^ HEDJ, DnaJ/Hsp-40-like genes, protein disulphide isomerise P5 (PDI-P5) and ribosome-associated membrane protein 4 (RAMP4) [130]. Different studies have shown that activation of IRE1 signalling is robust at first but as time progresses it diminishes [60,130,140]. However, artificial maintenance of IRE1 signaling is achieved by a chemically activated mutant form of IRE1 which positively correlated with enhanced cell survival conditions under ER stress; this provides an indication that IRE1 signaling mainly plays a role as a pro-survival pathway [37,130,140].

***ATF6***: ATF6 is a type II transmembrane protein that contains a basic leucine zipper (bZIP) transcription factor domain in its cytosolic terminus [130,131,141]. The ATF6 family of ER transducers includes ATF6α, ATF6β, old astrocyte specifically induced substance (OASIS), LUMAN (also called CREB3), BBF2 human homolog on chromosome 7 (BBF2H7), cyclic-AMP responsive element binding protein hepatocyte (CREBH) and CREBP4 [139]. Unlike IRE1, ATF6 does not undergo oligomerization, dimerization, and autophosphorylation. Under ER stress conditions, Grp78 dissociates from ATF6, thus uncovering the ATF6 Golgi localization signal. Activated ATF6 translocates into the Golgi complex where it undergoes cleavage by site-1 and site-2 proteases [133,137,139]. Thus, the N-terminal cleavage product of ATF6 translocates to the nucleus and regulates the expression of genes that are associated with the ER-associated protein degradation pathway. Some of the ATF6 target genes include Grp78, protein disulphide isomerise (PDI) and ER-degradation enhancing-a-mannosidase-like protein 1 (EDEM1). All these proteins work closely in concert to reduce unfolded proteins in the ER lumen [130]. ATF6 also activates pro-survival transcription factor and IRE1 target gene XBP1 [142]. Similar to that of IRE1 signaling, ATF6 is activated upon UPR but is not sustained throughout the UPR response. ATF6 signaling is primarily for pro-survival, but in some cases, it activates pro-apoptotic transcription factor C/EBP homologous protein (CHOP) during prolonged ER stress [142].

***PERK***: Protein kinase RNA-activated like ER kinase (PERK) is a type I ER transmembrane protein having an ER luminar sensor domain and a cytoplasmic domain. The cytoplasmic domain contains Ser/Thr kinase activity. Upon activation by UPR, PERK dissociates itself from Grp78 and undergoes dimerization and trans-autophosphorylation [130,131]. Activated PERK phosphorylates eukaryotic translation initiation factor 2α (eIF2α). PERK-mediated phosphorylation of eIF2α at Ser51 reduces the activity of the eIF2α complex and leads to the inhibition of protein synthesis. This rapidly reduces the number of proteins entering the ER and this can lead to pro-survival effects on the cell [133,141]. Phosphorylation of eIF2α also allows translation of mRNAs containing short open reading frames in their 5ʹ UTR regions. Such translated proteins include activating transcription factor 4 (ATF4) [133]. ATF4 controls the expression of many proteins involved in redox processes and amino acid metabolism. ATF4 also modulates the expression of ER chaperones and foldases [133,141]. ATF4 also regulates important genes involved in ER apoptosis such as CHOP and growth arrest and DNA damage-inducible 34 (GADD34) [133]. GADD34 is involved in a feedback loop to dephosphorylate eIF2α by protein phosphatase IC (PPIC) to restore protein synthesis [133,143]. Another substrate for activated PERK is a nuclear factor (erythroid-derived 2 factor)-related factor (Nrf2). In normal cells, Nrf2 is present in the cytoplasm in association with cytoskeletal anchor kelch-like Ech-associated protein (KEAP1). Upon activation, PERK phosphorylates Nrf2 and this helps Nrf2 to dissociate from KEAP1 and translocate into the nucleus [130,144]. Upon translocation into the nucleus Nrf2 induces the expression of genes with an anti-oxidant response element (ARE) within their promoter such as heme oxygenase 1 (HO-1) aiding in protein folding and helping to restore ER homeostasis [130,144]. The role of Nrf2 as a pro-survival factor is further proved by the fact that cells devoid of Nrf2 upon ER stress displayed increased sensitivity to cell death by means of apoptosis [130,144,145].

## Arboviruses and autophagy

Autophagy is associated with replication/translation of different arboviruses [146–149]. Several arboviruses invoke autophagy components such as the autophagosome, amphisome, and autolysosome not only to serve as a scaffold for viral replication, but also to escape from the immune system [147,150–152].

JEV infection induces autophagy in several cell types. A study which used an Atg5/Beclin-1 knock down model and monitored LC3 lipidation in JEV-infected NT-2 cells, a pluripotent human testicular embryonal carcinoma cell line, after treatment with rapamycin and 3-methyladenine, revealed a direct relationship between autophagy and viral replication. They showed that autophagy has supporting roles in JEV replication in the early stages of infection [147]. However, there is a contrasting view of the cross-talk between JEV and autophagy. Sharma et al. observed in their study, using Neuro2a cells, and WT and atg5−/− mouse embryonic fibroblast (MEFs), that autophagy primarily restricts viral replication. They observed significant colocalization between NS1 and EDEM1 and that autophagy antagonizes JEV infection and functions to limit viral replication and reduce viral yields through LC3-I- and EDEM1-containing membranes [153].

The replication of DENV is positively linked to autophagy induction by which the initiation of autophagy is enhanced in DENV-infected cells [154,155]. However, DENV replication is cell-specific and it would be limited in monocytes [156]. Regarding WNV, which is a neurotropic flavivirus responsible for meningitis and encephalitis, its replication is autophagy independent; however, it still induced autophagy in different mammalian cell lines [157]. Some evidence supports upregulation of autophagy by WNV [158,159], whereas some other evidence does not [157]. A study by Blázquez and colleagues mapped the genetic determinants of autophagy regulation in WNV-infected Vero cells and thus clarified the controversy concerning the induction of autophagic responses in WNV-infected cells [160]. They highlighted that amino acid substitutions in the viral non-structural proteins 4A or 4B can modulate the induction of autophagy in WNV-infected cells independently of the activation of the unfolded protein response [160]. Another study showed that JEV infection of neuronal cells activated all three pathways of ER stress (UPR). However, they showed that a crucial link exists between two ER stress pathways (XBP1 and ATF6) and autophagy in JEV-infected neuronal cells [161].

**Figure 1.**
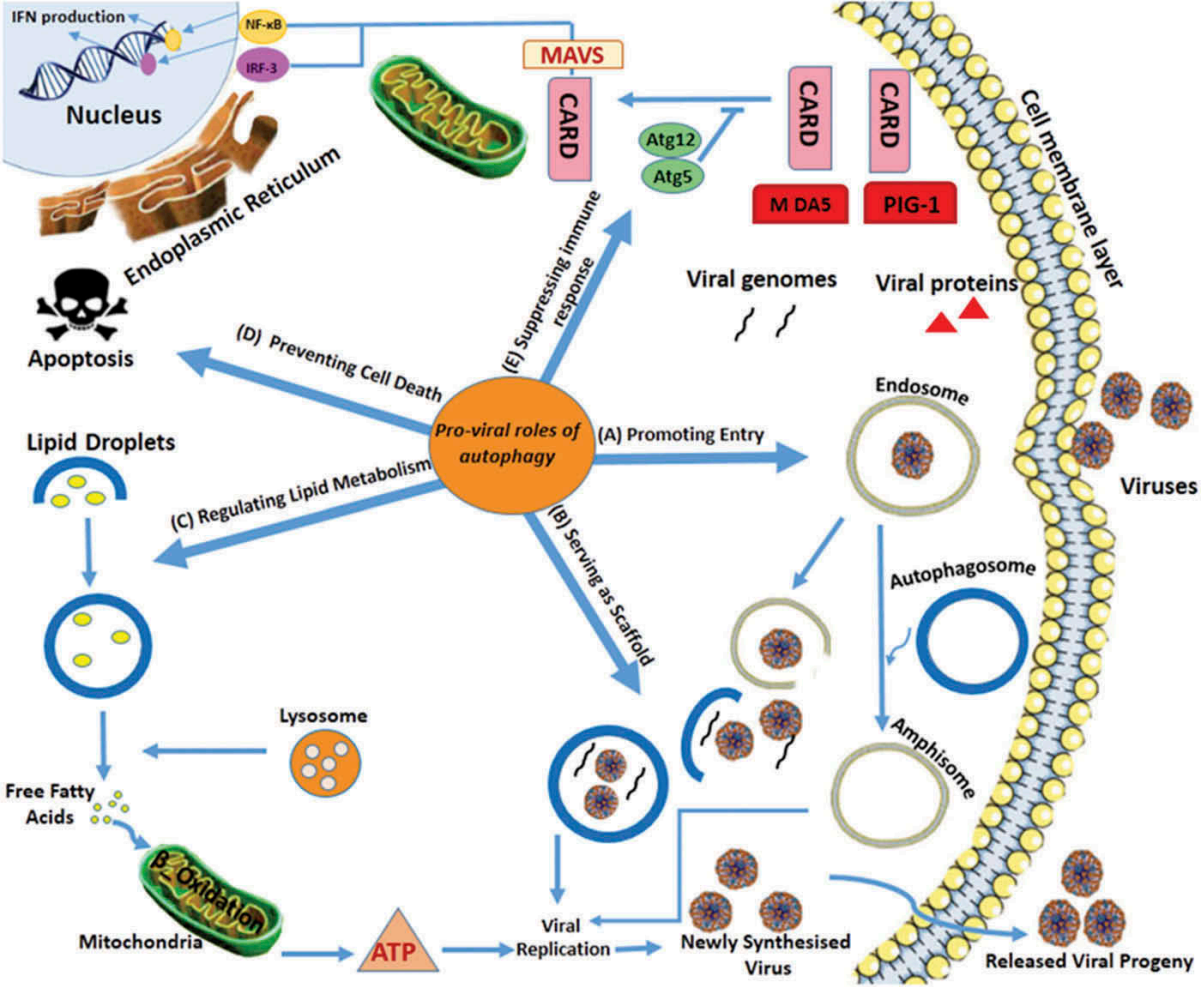
Autophagy Signaling During Arbovirus Infection. There are five possible mechanisms for modulating viral replication which include: a) some arboviruses such as DENV and JEV can use amphisome formation for their entry and replication; b) several arboviruses such as DENV, ZIKV, JEV, CHIKV and TBEV exert diverse mechanisms to induce autophagosome formation to enhance viral replication/translation complexes. DENV is associated with NS4A in up-regulating PI3K-dependent autophagy. CHIKV induces the IRE1α–XBP-1 pathway in conjunction with ROS-mediated mTOR inhibition; c) DENV benefits from autophagy activation by using lipid droplets as an energy source for replication; d) Viruses such as DENV-2 and CHIKV can increase their replication by prolonging cell survival and preventing cell death; and d) VSV appears to suppress IFN signaling by conjugated Atg5-Atg12, leading to an effective virus-suppressing immune response [modified from [131]] . DENV: Dengue virus; ZIKV: Zika virus; JEV: Japanese encephalitis virus; CHIKV: Chikungunya virus; TBEV: tick-borne encephalitis virus; VSV: vesicular stomatitis virus.

The relationship between up-regulation of viral replication and virus-induced autophagy also has been shown in Chikungunya virus (CHIKV)-infected HEK293 (human embryonic kidney) cells [148]. Epizootic hemorrhagic disease virus (EHDV) induces autophagy, apoptosis, activates c-Jun N-terminal kinase (JNK) and phosphorylates c-Jun, all of which benefit EHDV replication [149]. Lee and colleagues suggest that DENV2-related pathogenesis in suckling mice were enhanced by autophagy, possibly by promoting viral replication whereas survival rate was reduced upon autophagy stimulation [162]. The amphisomes play major roles in DENV entry and translation/replication [152]. DENV-2 interacts with amphisomes while DENV-3 interacts with both amphisomes and autophagolysosomes for their translation/replication (Figure 1) [152]. CHIKV also increases autophagosome formation as a site for aggregation of viral translation/replication complexes [148,163]. These studies have shown the presence of viral replication/translation complexes of DENV and JEV in both the autophagosome and the endosome, which suggests an auxiliary role for autophagosome–endosome fusion in viral entry [147,164]. Although the exact mechanism of autophagosome accumulation in JEV replication is not clear yet, some studies have suggested the importance of autophagy in reducing mitochondrial antiviral signaling protein (MAVS)-IRF3 activation to facilitate virus replication [165]. It has been suggested that during JEV infection, the autophagy process promotes cell survival by delivering damaged mitochondria to lysosomes [165]. The DENV2 non-structural viral protein NS4A has been characterized as a main component of the DENV2 replication complexes [166]. This virus’ replication/translation is associated with NS4A in up-regulating PI3K-dependent autophagy, and preventing cell death [167]. In regards to CHIKV, it has been demonstrated that autophagy postpones apoptosis and promotes CHIKV propagation by inducing the IRE1α–XBP-1 pathway in conjunction with ROS-mediated mTOR inhibition (Figure 1) [168]. In relation to DENV, lipid droplet usage as an energy source is an autophagy-mediated pro-viral mechanism that is used for this virus’ replication [128]. There are several miRNAs produced during persistent infection of mosquito cells with DENV, which help to regulate proteins that participate in processes such as autophagy [169]. A study on vesicular stomatitis virus (VSV), showed that the interaction of PRR-PAMP with MAVS through caspase recruitment domains (CARDs) of retinoic acid-inducible gene-I (RIG-I) and melanoma differentiation associated gene 5 (MDA5) by homotypic reaction, leads to signaling cascades that ultimately activate nuclear factor-κB (NF-κB) and interferon regulatory factors (IRF-3) [121,170]. Inhibiting IFN production followed by interaction of atg5-atg12 with the CARDs of RIG-I and MDA5 inhibits IFN-β promoter stimulator 1 (IPS-1), which can promote VSV replication (Figure 1) [171].

Several studies were conducted on ZIKV. The results of the study by Hamel et al., showed a major role for the phosphatidylserine receptor AXL as a ZIKV entry receptor, and cellular autophagy in enhancing ZIKV replication in permissive cells [172]. Thus, ZIKV is able to increase its replication via induction of autophagy in infected skin fibroblasts. A murine experimental model was infected with Brazilian ZIKV. It was demonstrated that Brazilian ZIKV crosses the placenta and causes microcephaly by targeting cortical progenitor cells, inducing cell death by autophagy and apoptosis in mouse neural tissue, and impairing neurodevelopment [173]. Although Drosophila is a model organism that does not recapitulate the unique physiological and anatomical environment associated with mosquitoes [174], a study on the Drosophila brain system suggested an essential role for Drosophila stimulator of interferon genes (dSTING)-dependent autophagy to restrict ZIKV infection and to control neuronal infection [175].

Tick-borne encephalitis virus (TBEV), which is an important travel-associated arbovirus, replicates in neural cells, inducing neuronal dysfunction, membrane rearrangements and autophagosome formation [176,177]. A schematic representation and a summary of findings about autophagy and arbovirus replication are summarized in Figure 1 and Table 1.

**Table 1.** Summary of arbovirus and autophagy pathway.

Reference	Summary of the study	Conclusions
^[147]^	Studied Atg5/Beclin-1 knock down model, monitored LC3 lipidation in JEV-infected NT-2 cells	Revealed a direct relationship between autophagy and JEV replication
^[165]^	Studied JEV infection	Autophagy process promotes cell survival by delivering damaged mitochondria to lysosomes Autophagy reduces MAVS-IRF3 activation to facilitate virus replication
^[156]^	Studied the replication of DENV	DENV replication is cell-specific and it would be limited in monocytes
^[166]^		NS4A protein has been characterized as a main component of the DENV2 replication complexes
^[167]^		DENV replication/translation is associated with NS4A in up-regulating PI3K-dependent autophagy, and preventing cell death
^[169]^		miRNAs help to regulate the proteins that participate in autophagy during persistent infection of mosquito cells with DENV
^[157]^	Studied WNV replication	DENV replication was shown to be autophagy independent; however, it still induced autophagy
^[158,159]^		supported upregulation of autophagy by WNV
^[160]^	mapped the genetic determinants of autophagy regulation in WNV infected cells	Amino acid substitutions in the NS 4A or 4B proteins can modulate the induction of autophagy in WNV-infected cells
^[148]^	Studied CHIKV replication	Showed the relation between up-regulation of viral replication and virus-induced autophagy in CHIKV-infected cells
^[148,163]^		CHIKV also increases autophagosome formation as a site for aggregation of viral translation/replication complexes
^[168]^		Autophagy postpones apoptosis and promotes CHIKV propagation by inducing the IRE1α–XBP-1 pathway in conjunction with ROS-mediated mTOR inhibition
^[149]^	Studied EHDV replication	EHDV induces autophagy, apoptosis and activates c-Jun N-terminal kinase (JNK) and phosphorylates c-Jun which all benefit its replication
^[162]^	Studied autophagy in DENV2-infected suckling mice	DENV2-related pathogenesis and survival rate of the suckling mice were enhanced by autophagy, possibly by promoting viral replication
^[152]^	Studied DENV-2 and −3 replication	DENV-2 interacts with amphisomes while DENV-3 interacts with both amphisomes and autophagolysosomes
^[171]^	Studied VSV replication	Inhibiting IFN production followed by interaction of atg5-atg12 with the CARD of RIG, and MDA5 can promote VSV replication
^[172]^	Studied ZIKV replication	Showed a major role for the phosphatidylserine receptor AXL as a ZIKV entry receptor, and cellular autophagy in enhancing ZIKV replication in permissive cells
^[173]^	Used murine experimental model to infect with Brazillian ZIKV	ZIKV replication is enhanced via induction of autophagy in infected skin fibroblasts. It was demonstrated that Brazilian ZIKV crosses the placenta and causes microcephaly
^[175]^	Studied Drosophila brain system	Suggested an essential role for dSTING-dependent autophagy to restrict ZIKV infection and to control neuronal infection
^[176,177]^	Studied TBEV replication	TBEV infects and replicates in neural cells inducing neuronal dysfunction, membrane rearrangements and autophagosome formation

CHIKV: Chikungunya virus; DENV: Dengue virus; EHDV: Epizootic hemorrhagic disease virus; JEV: Japanese encephalitis virus; RVFV: Rift Valley fever virus; TBEV: Tick-borne encephalitis virus; VSV: Vesicular stomatitis virus; WNV: West Nile virus; ZIKV: Zika virus.

## Arboviruses and apoptosis

**Figure 2.**
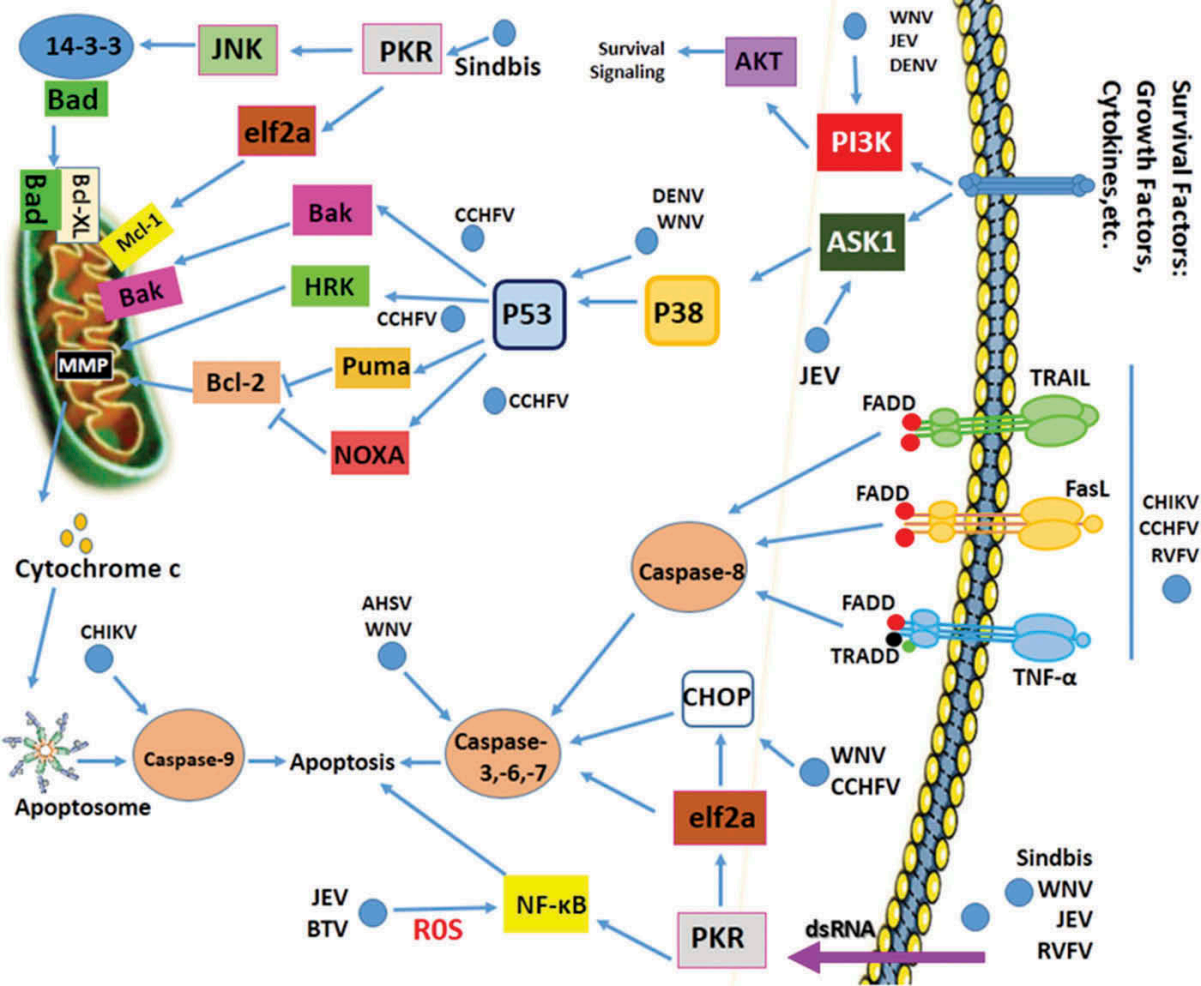
Apoptosis Signaling During Arbovirus Infection. Arboviruses exert their effect on apoptosis through different signaling routes. A mechanism for anti-apoptotic activity by these viruses is up-regulation of the PI3K signaling pathway. Another mechanism that viruses can regulate is the initiation of protein 14–3-3 through activation of JNK followed by induction of PKR. CCHFV replication is associated with upregulation of Bax, HRK, PUMA, and Noxa. WNV, JEV and DENV block or delay apoptosis via activating PI3K/Akt signaling. WNV can trigger apoptosis after several rounds of replication through caspases-3 and −12 and p53. JEV triggers ROS-mediated ASK1-ERK/p38 MAPK activation which leads to initiation of apoptosis. JEV may affect Bcl-2 expression to increase anti-apoptotic response. DENV may subvert apoptosis by inhibiting NF-kB. DENV reduces immune responses by activation of p53-dependent apoptosis. RVFV inhibits caspase-8 to regulate pro-apoptotic p53 signaling. The BTV-induced apoptosis involves NF-kB [modified from [131]]. DENV: Dengue virus; ZIKV: Zika virus; WNV: West Nile virus; JEV: Japanese encephalitis virus; CHIKV: Chikungunya virus; CCHFV: Crimean Congo hemorrhagic fever virus; RVFV: Rift Valley fever virus; BTV: bluetongue virus.

Arboviruses such as SINV, WNV, and JEV use apoptosis as a virulence factor to promote their pathogenesis [178–180]. Each of these viruses has its own-specific targets and biochemical-induced mechanisms during virus-induced programmed cell death. Observations suggest that apoptosis induced by SINV plays an important role in virus pathogenesis and mortality [178]. After the entry of SINV into host cells, dsRNA intermediates are formed, then dsRNA-dependent protein kinase (PKR) recognizes these particles [181–183]. PKR blocks cellular translation through eIF2α phosphorylation, which inhibits MCL-1 biosynthesis, an anti-apoptotic Bcl2 family protein [184]. PKR also controls c-Jun N-terminal kinases (JNK) through IRS1 phosphorylation and then activates 14–3-3 proteins (Figure 2). Thus, 14–3-3 proteins affect the accessibility of Bad to kinases and serves to localize kinases to their substrates, causing the release of Bad and disruption of the complex between anti-apoptotic Bcl2 family proteins, Bcl-xl and Bak. Both Bad and Bik can displace Bak from MCL-1, which results in Bak oligomerization and cytochrome C release, and subsequent induction of apoptosis in MOSEC tumor cells, derived from the ovarian epithelium, and Pan02, derived from a pancreatic adenocarcinoma [185]. CHIKV triggers the apoptosis pathway to evade the immune system and facilitate its dissemination by infecting neighboring cells [186]. CHIKV infection triggers both apoptosis and autophagy. However, based on kinetic studies, it was found that CHIKV-induced autophagy delays caspase-dependent apoptotic cell death by inducing the IRE1α-XBP-1 pathway in conjunction with ROS-mediated mTOR inhibition [168].

The replication of Crimean-Congo hemorrhagic fever virus (CCHFV), (family *Bunyaviridae*), is associated with the extracellular pathway of apoptosis. Up-regulation of pro-apoptotic proteins (i.e. Bax and HRK) and novel components of the ER stress-induced apoptotic pathways (i.e. PUMA and Noxa) have been shown in a CCHFV-infected hepatocyte cell line, which suggests a link between CCHFV replication, apoptosis and ER stress (Figure 2). Notably, the differential high levels of CHOP, a transcription factor which is activated through ER stress, are present in hepatocyte cells following CCHFV replication [187]. During CCHFV infection the over-expression of IL-8, which is an apoptosis inhibitor, was independent from apoptotic pathways. However, another investigation showed a positive relationship between IL-8 induction and DENV infection [187–189]. In contrast to SINV, CHIKV and CCHFV replication in infected cells are necessary for apoptosis induction, which was demonstrated by UV-inactivated viral particles [190–192]. A proteomic analysis of CHIKV-infected astrocytic cells provided a comprehensive spectrum of modulated host proteins. This study showed that Nucleophosmin (NPM1)/B23, a nucleolar multifunctional chaperone, plays a critical role in restricting CHIKV replication [193]. Studies of mouse models comparing the East Central South African (ECSA) and Asian strains of CHIKV have shown that both strains can spread to astrocytes and neurons; however, studies with the Asian strain showed increased expression of pro-apoptotic genes and higher mortality [194].

Flavivirus replication (e.g. WNV, JEV and DENV) can be limited by virus-induced programmed cell death at the early stage of virus infection. These viruses may block or delay apoptosis by activating PI3K/Akt signaling which improves their replication rate (Figure 2) [190,195]. Blocking PI3K in neuronal N18 cells (using LY294002 or wortmannin) showed that apoptosis induction might be due to p38 MAPK activation and did not affect JEV and DENV viral particle production [190]. In 2001, it was demonstrated for the first time that WNV-induced cytopathic effect was caused during the induction of apoptosis. It was also found that viral replication is essential for virus-induced cell death in K562 and Neuro-2a cells [196]. The WNV capsid protein has anti-apoptotic functions which can block or delay apoptosis by suppression of the PI3-kinase-dependent process at the early stage of infection [195]. Akt, which is a downstream target of PI3-kinase, can directly phosphorylate Bad at position Ser136 [197]. However, WNV can trigger apoptosis after several rounds of replication through caspases-3 and −12 and p53 and it is important to note that the initial viral dose affects the kinetics of WNV-induced cell death [191,198–200]. Replication of several RNA viruses might be affected by the expression of multiple miRNAs in host cells either positively or negatively. One such miRNA is Hs_154, which limits WNV replication in HEK293 and SK-N-MC cells by inhibition of two anti-apoptotic proteins; CCCTC binding factor (CTCF), and EGFR-co-amplified and overexpressed protein (ECOP) [190,201]. JEV is an RNA virus which may trigger ROS-mediated ASK1-ERK/p38 MAPK activation, which leads to initiation of apoptosis (Figure 2) [202]. In another experiment, mouse neuroblastoma cell line N18 was infected with UV-inactivated JEV (UV-JEV). These dead virions induced cell death through a ROS-dependent and NF-kB-mediated pathway [203]. The initial suppression of UV-JEV-induced cell death, followed by co-infection with active or inactive JEV, demonstrated that JEV may trigger cell survival signaling in mouse neuroblastoma N18 and human neuronal NT-2 cells to modify cellular pathways for timely virus production [203]. NS1‘ protein, a neuroinvasiveness factor that is produced by the JEV serogroup of Flaviviruses, was introduced as a caspase substrate; however, using a caspase inhibitor had no effect on virus replication [204]. The experimental evidence showed that JEV may affect Bcl-2 expression to increase anti-apoptotic response to enhance virus persistence and reach a balance between cell death and virus replication [205]. JEV may also enhance blood–brain barrier permeability through up-regulation of Bax, Bid, Fas and FasL and down-regulation of IGFBP-2, Bid, p27 and p53 [206]. The results from a macaque model study indicated neuronal apoptotic death along with the release of pro-inflammatory cytokines which are crucial steps in the pathogenesis of JEV [207].

Several studies have confirmed the effect of DENV on apoptosis in a wide variety of mammalian cells including hepatocytes, monocytes, endothelial cells, dendritic cells, mast cells, and neuroblastoma cells. However, the mechanisms are not yet fully understood. Dendritic cells are believed to be the primary targets for DENV and play central roles in supporting active replication for virus pathogenesis. However, a study reported that replication of DENV in monocyte-derived dendritic cells (mdDCs) was positively correlated with TNFα and apoptosis [208]. To achieve high rates of replication, DENV may subvert apoptosis in macrophages, hepatoma, and dendritic cells, by inhibiting NF-kB in response to TNFα stimulation [201,209]. DENV replication was positively affected by inhibiting apoptosis by the interaction between capsid protein and the hepatoma cell line (Huh7) calcium modulating cyclophilin-binding ligand (CAML) [201]. Activation of p53-dependent apoptosis by DENV may contribute to the inhibition of inflammation and reduction of immune responses to efficiently disseminate viral progeny (Figure 2). This research was conducted on a p53-deficient cell line, H1299, and on a p53-knockin cell line [210]. Following DENV infection in these cell lines, a microarray analysis revealed that activation of the pro-apoptotic gene caspase-1 played a basic role in the p53-mediated apoptotic pathway and was necessary for up-regulation of different immune response genes [210]. The WNV capsid protein was shown to be capable of inducing the p53-dependent apoptotic process in wild-type mouse embryonic fibroblasts (MEF) or SH-SY5Y cells. It showed no significant effects on p53-null MEF or on p53-knockdown SH-SY5Y cells [199]. The pro-apoptotic NSs and anti-apoptotic NSm proteins of the Phelebovirus genus of the family *Bunyaviridae* (e.g. RVFV) delayed apoptosis in Human small airway lung epithelial cells (HSAECs) to efficiently replicate virus by regulating p53 and favor virus propagation [211]. RVFV infection in Vero E6 and HEK293 cells inhibits either caspase-8 or the extracellular apoptotic pathway to regulate pro-apoptotic p53 signaling (Figure 2) [212]. The viral NSs protein can facilitate viral translation through inhibition of the PKR/eIF2α pathway and blockage of IFN at early stages of infection [213]. The genus Orthobunyavirus of the family *Bunyaviridae* delayed apoptosis in a cell line (P2.1), derived from U4C, defective in double-stranded RNA signaling due to low levels of IRF-3 through anti-apoptotic effects of NSs protein on IRF-3 activity [214].

*Reoviridae* replication is extensively linked to apoptosis. Bluetongue Virus (BTV), a member of this family, induces apoptosis in three studied mammalian cell lines (HeLa (human cervical epithelial carcinoma), BSR (baby hamster kidney), and HEK293T) but not in insect cell lines. Apoptosis induced by BTV involved activation of NF-kB, which required virus uncoating and exposure to outer capsid proteins VP2 and VP5 (Figure 2) [215]. African horse sickness virus (AHSV) is another arbovirus which also induced apoptosis in mammalian BHK-21 cells but not in insect KC cells, through activation of caspase-3 [216].

A histopathological analysis has reported DENV-2 infection in livers of BALB/c mice. Necrosis and apoptosis were clearly noticed as cytopathic effects of DENV-2 infection [217]. The ability to induce apoptosis following targeted splicing of viral genomes is an important advantage of this antiviral approach. Carter and colleagues devised a unique configuration of anti-CHIKV/DENV dual targeting group I intron, which catalyzes trans-splicing of the 5ʹ conserved target sequences of the DENV and CHIKV genomes to a 3ʹΔN Bax exon, to effectively induce apoptotic cell death in *Aedes albopictus* C6/36 cells following infection, thus preventing viral spread [218]. Another study on this cell line used synthetic miRNAs to induce dual DENV-3/CHIKV-resistance phenotypes in the vector mosquito *Aedes aegypti* using transgenic mosquitoes which were generated using Class II TE mariner MosI. They targeted the conserved DENV and CHIKV sequences, which then could lead to viral RNA trans-splicing and cell apoptosis [219].

ZIKV infection in epidermal keratinocytes caused the appearance of cytoplasmic vacuolation, and the presence of pyknotic nuclei in the stratum granulosum, which is indicative of apoptotic cells [172]. The South Pacific epidemic strain of ZIKV (PF-25,013–18) can replicate in A549 cells. This infection enhanced Type-I IFNs, ISGs, pro-inflammatory cytokines and delayed mitochondrial apoptosis [220]. ZIKV, which infects neural progenitor cells in organoid and neurosphere models, activates Toll-like receptor 3 which triggers apoptosis and attenuates neurogenesis [221]. Different strains of ZIKV make use of different structural proteins which affects the permissiveness of human epithelial and neuronal cells to infection by this virus [222]. Saint Louis encephalitis virus (SLEV) is a neglected flavivirus. Its epidemic strain, CbaAr-4005, was studied and the results indicated probable entrance of SLEV to the CNS from the circulatory system. Thus, severe disorders could be induced by this infection within the CNS of infected mice. Neuropathogenesis induced by SLEV in mice was screened using different types of neuronal degeneration, and a significant increase in the number of apoptotic cells in infected mice compared to uninfected ones was confirmed [223].

Until recently, the role of apoptosis in determining the outcome of arbovirus infection in mosquitoes was not clear; however, to find a correlation between apoptosis and arbovirus infection, it is reasonable to also study this pathway in the invertebrate mosquito vector. A study for the first time tested the roles of apoptosis and caspases directly in determining mosquito vector competence for arboviruses infection using the SINV model. Silencing of the *A. aegypti* anti-apoptotic gene iap1 (Aeiap1) in adult female *A. aegypti* mosquitoes caused apoptosis in midgut epithelium, and enhanced mosquito mortality and susceptibility to SINV infection. However, silencing of initiator caspase gene, Aedronc, protected mosquitoes against mortality and reduced SINV midgut infection [224]. Arboviruses have evolved mechanisms to avoid apoptosis in mosquito vectors. Mosquitoes were infected with SINV that expressed a proapoptotic gene, Reaper. The Reaper-expressing virus showed replication defects in mosquitoes [225]. *A. albopictus* also is a vector of various arboviruses. Aadnr1, a novel gene related to innate immunity and apoptosis in *A. albopictus*, an ortholog of dnr1 in Drosophila, was studied in C6/36 mosquito cells. Aadnr1 encodes a protein that contains an N-terminal FERM domain and a C-terminal RING domain which are involved in signal transduction pathways and ubiquitination, respectively. The transcriptional level of Aadnr1 and subsequently apoptosis were reduced after SINV infection [62]. An effector caspase, AaCASPS7, in *A. albopictus* also induced caspase-dependent apoptosis in C6/36 cells [226], which could indicate an apoptotic caspase in arbovirus infection. Thus, apoptosis could be considered one of the defense pathways in mosquitoes against arbovirus infections, and is probably a factor to determine vector competence [227]. A study by Troupin et al. showed that mosquito ubiquitin Ub3881 in an Aag2 *A. aegypti* cell line plays roles in apoptosis of the mosquito cells during DENV infection. Ub3881 overexpression targeted DENV envelope protein and reduced virion production. The loss of Ub3881 function reduced the level of apoptosis during DENV infection [228]. This suggests it would be worthwhile to test Ub3881 peptides as inhibitors of DENV infection in mosquitoes.

As indicated above, several studies have examined the interaction between arboviruses and apoptosis pathways; however, the exact mechanisms whereby arboviruses modulate apoptosis need to be more extensively studied. A schematic representation and a summary of findings about apoptosis and arbovirus replication are summarized in Figure 2 and Table 2.

**Table 2.** Summary of arbovirus and apoptosis pathway.

Reference	Summary of the study	Conclusions
^[178]^	Studied SINV replication	Apoptosis induced by SINV plays an important role in virus pathogenesis and mortality
^[181–183]^		After the entry of SINV into the host cell the dsRNA intermediates are formed, then dsRNA-dependent protein kinase (PKR) recognizes these particles
^[186]^	Studied CHIKV replication	The CHIKV triggers the apoptosis to evade immune system and facilitate its dissemination by infecting neighboring cells
^[168]^		CHIKV infection can induce apoptotic cell death via intrinsic and extrinsic pathways and facilitates virus release and spread
^[193]^	The proteomic analysis in astrocytic cells infected with CHIKV	It showed that Nucleophosmin (NPM1)/B23, a nucleolar multifunctional chaperone, plays a critical role in restricting CHIKV replication
^[194]^	Studied mouse models comparing African and Asian strains of CHIKV	Both strains can spread to astrocytes and neurons, however, those with the Asian strain showed increased expression of pro-apoptotic genes and higher mortality
^[187–189]^^[190]–192^	Studied apoptosis by UV-inactivated viral particles	During CCHFV infection, the over-expression of IL-8 which is an apoptosis inhibitor was independent from apoptotic pathways. CCHFV replication is necessary for apoptosis induction, which was demonstrated by UV-inactivated viral particles
^[190]^^[217]^	A histopathological analysis about DENV-2 infection on liver of BALB/c mice	Blocking PI3K showed that apoptosis induction might be due to p38 MAPK activation and did not affect JEV and DENV viral particle production Necrosis and apoptosis were clearly noticed as cytopathic effects of DENV-2 infection
^[197]^	Studied WNV replication	Akt can directly phosphorylate Bad at position Ser 136 in WNV infected cells
^[190,201]^	Studied expression of multiple miRNAs in JEV infection	One miRNA, Hs_154, limits WNV replication by inhibition of two anti-apoptotic proteins like CCCTC binding factor (CTCF) and EGFR-co-amplified and overexpressed protein (ECOP)
^[203]^	Studied JEV in mouse neuroblastoma cell line N18	N18 was infected with UV-inactivated JEV (UV-JEV). These virions induced cell death through a ROS-dependent and NF-kB-mediated pathway
^[203]^	Studied JEV replication	The initial suppression of UV-JEV-induced cell death, followed by co-infection with active or inactive JEV, demonstrated that JEV may trigger cell survival signaling to modify cellular pathways for timely virus production
^[204]^		NS1‘ protein, a neuroinvasiveness factor which is produced by the JEV, was introduced as a caspase substrate; however, using a caspase inhibitor had no effect on virus replication
^[206]^		JEV may enhance blood–brain barrier permeability through up-regulation of Bax, Bid, Fas and FasL and down-regulation of IGFBP-2, Bid, p27 and p53
^[207]^	Studied JEV in JEV macaque model	The results from a macaque model study indicated neuronal apoptotic death along with the release of pro-inflammatory cytokines which are crucial steps in the pathogenesis of JEV
^[208]^	Studied DENV replication	Replication of DENV in monocyte-derived dendritic cells was positively correlated with TNFα and apoptosis
^[210]^		Activation of the pro-apoptotic gene caspase-1 played a role in p53-mediated apoptotic pathway and was necessary for up-regulation of different immune response genes following DENV infection
^[210]^	microarray analysis	
^[199,211]^	Studied RVFV replication	NSs and NSm proteins of RVFV delayed apoptosis to efficiently replicate by regulating p53
^[213]^		The NSs protein can facilitate viral translation through inhibition of PKR/eIF2α pathway and production of IFN at early stages of infection
^[216]^	Studied AHSV replication	AHSV induced apoptosis in mammalian BHK-21 cells through activation of caspase-3
^[218]^	Devised anti-CHIKV/DENV dual targeting group I intron	Effectively induced apoptotic cell death following infection, thus preventing viral spread
^[219]^	synthesized miRNAs to induce dual DENV-3/CHIKV -resistance phenotypes in the vector mosquito Aedes aegypti	Targeted the conserved DENV and CHIKV sequences, which then led to viral RNA trans-splicing and cell apoptosis
^[172]^	Studied on ZIKV infection	ZIKV infection in epidermal keratinocytes caused the appearance of cytoplasmic vacuolation, and the presence of pyknotic nuclei in the stratum granulosum, which is indicative for apoptotic cells
^[220]^		South Pacific epidemic strain of ZIKV (PF-25,013–18) can replicate in A549 cells. This infection enhanced Type-I IFNs, ISGs, pro-inflammatory cytokines and delayed mitochondrial apoptosis
^[221]^		ZIKV which infects neural progenitor cells in organoid and neurosphere models, activates Toll-like receptor 3 which triggers apoptosis and attenuates neurogenesis
^[222]^		Different strains of ZIKV take use of different structural proteins which affect the permissiveness of human epithelial and neuronal cells to this infection
^[223]^	Studied on SLEV; its epidemic strain; CbaAr-4005	Suggested probable entrance of SLEV to the CNS from the circulatory system, thus severe disorders induction by this infection within the central nervous system of infected mice
^[224]^	Silencing of the A. aegypti anti-apoptotic gene iap1 (Aeiap1) and silencing of initiator caspase gene, Aedronc in adult female A. aegypti mosquitoes	Silencing of the Aeiap1 caused apoptosis in midgut epithelium, and enhanced mosquito mortality and susceptibility to SINV infection. However, silencing of Aedronc protected mosquitoes against mortality and reduced SINV midgut infection
^[225]^	Mosquitoes were infected with SINV that expressed a proapoptotic gene, Reaper.	The Reaper-expressing virus showed replication defects in mosquitoes
^[62]^	The Aadnr1, a novel gene related to innate immunity and apoptosis in *Aedes albopictus*, ortholog of dnr1 in Drosophila, was studied in C6/36 mosquito cells	After infection with SINV the transcriptional level of Aadnr1 and subsequently the apoptosis were reduced AaCASPS7 induced caspase-dependent apoptosis in C6/36 cells in Aedes albopictus
^[226]^^[227]^	Studied an effector caspase; AaCASPS7	AaCASPS7 could be indicated as an apoptotic caspase in arbovirus infection. Thus, apoptosis could be considered as one of the defense pathways in mosquitoes against arbovirus infections, and is probably a factor to determine vector competence
^[228]^	Studied mosquito ubiquitin Ub3881	Ub3881 plays role in apoptosis of the mosquito cells during DENV infection. The Ub3881 overexpression targeted DENV envelope protein and reduced virion production. The loss of Ub3881 function reduced the level of apoptosis during DENV infection

AHSV: African horse sickness virus; CCHFV: Crimean Congo hemorrhagic fever virus; CHIKV: Chikungunya virus; DENV: Dengue virus; EHDV: Epizootic hemorrhagic disease virus; JEV: Japanese encephalitis virus; RVFV: Rift Valley fever virus; SINV: Sindbis virus; SLEV: Saint Louis encephalitis virus; TBEV: Tick-borne encephalitis virus; VSV: Vesicular stomatitis virus; WNV: West Nile virus; ZIKV: Zika virus.

## Arboviruses and UPR

**Figure 3.**
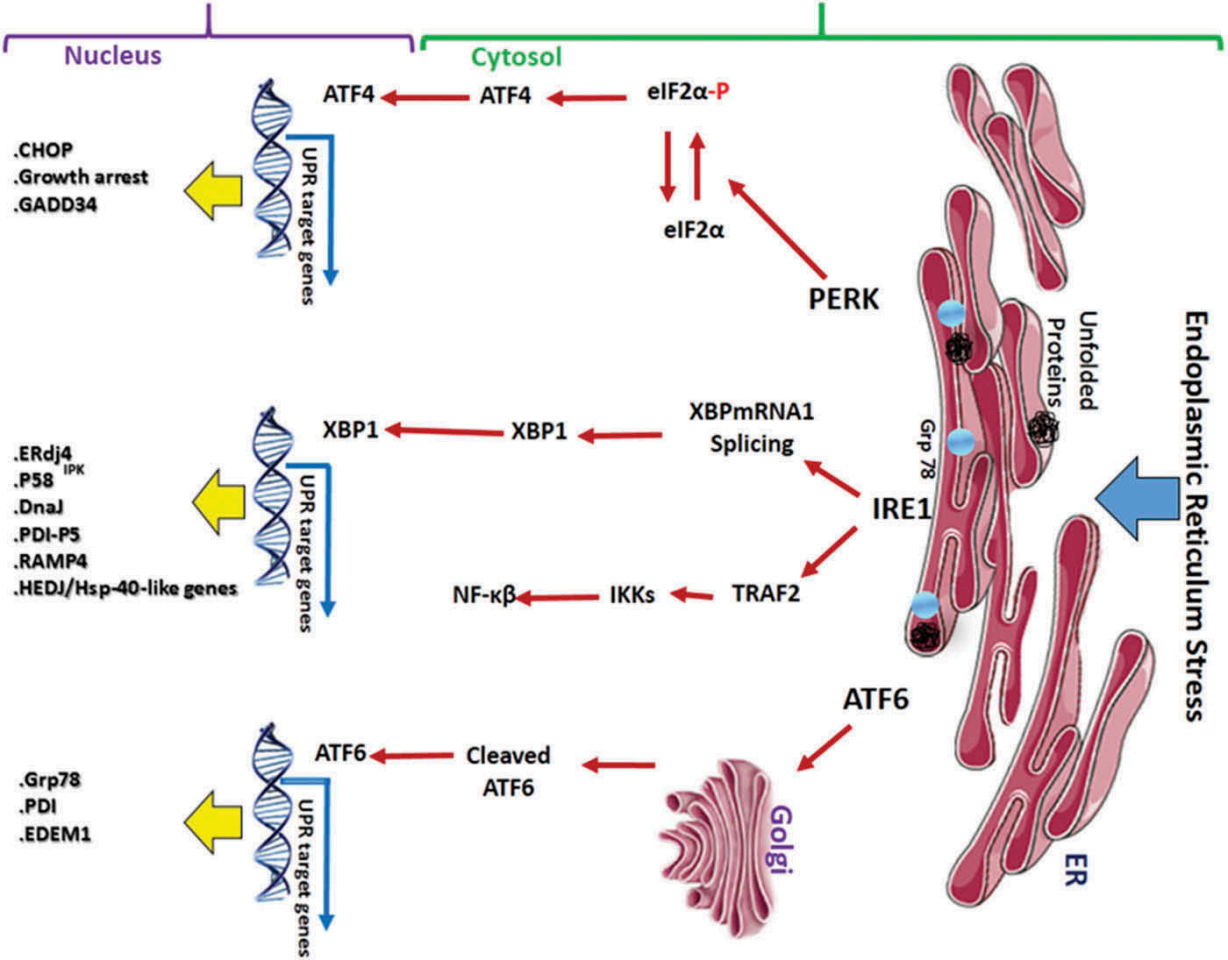
UPR Signaling During Arbovirus Infection. ER stress is enhanced in viral infected cells and activates UPR proteins (e.g. PERK, ATF6, and IRE1). Activated PERK induces ATF4 via phosphorylation of eIF2α, causing attenuation of translation and genes encoding CHOP. Upon IRE1 activation, TRAF2 and XBP mRNA1 splicing are initiated in the cytoplasm, which subsequently leads to regulation of UPR target genes. The degradation of ATF6 is increased through recruitment of ATF6, a UPR sensor, which results in the regulation of protein folding. The consequences of UPR activation are necessary for viral replication and pathogenesis [modified from [131]].

WNV activates multiple UPR pathways which cause activation of several UPR target genes [200]. The XBP1 pathway was shown not necessary for WNV replication; however, ATF6 was degraded by the proteasome and the PERK pathway transiently phosphorylated eIF2α and induced the pro-apoptotic protein CHOP (Figure 3) [200]. Cells infected with WNV showed signs of apoptosis including induction of growth arrest and activation of caspase-3 and PARP. The WNV titer was also significantly increased in a CHOP^−/-^ deficient MEF cell line but not in wild type MEF cells [200]. The host mechanism to counteract WNV infection involved activation of CHOP-dependent cell death [200]. The evidence confirmed the activation of UPR in WNV infection via ATF6/IRE1 pathways [200,229]. However, there was no significant phosphorylation of eIF2α, indicating that the UPR PERK pathway was not activated [229]. The WNV Kunjin strain NS4A and NA4B proteins were recognized as potent inducers of UPR. Moreover, sequential removal of NS4A hydrophobic domains decreased UPR activation but increased IFN-γ-mediated signaling in Vero C1008 cells [229]. These results showed that hydrophobic residues of WNV Kunjin strain NS proteins activate UPR signaling. The results from the same study group showed that ATF6 signaling is required for WNV replication by promoting cell survival and innate immune response inhibition [230]. The ATF6-deficient cells showed a decrease in WNV Kunjin strain protein and virion production. These cells also demonstrated increased eIF2α phosphorylation and CHOP transcription, which was absent in infected control cells [230]. In contrast, upon infection with WNV, IREI-deficient cells did not show any distinct differences as compared to IREI-positive cells [230]. The results also indicated the essential role of ATF6 for viral replication. In the absence of ATF6, other UPR signaling cascades such as the PERK and IRE1 pathways could not activate or enhance virus production. It was also shown that both ATF6 and IREI are required for STATI phosphorylation, highlighting the necessity of ATF6 for inhibition of innate immune response [230]. CHIKV and SINV also cause frequent epidemics of febrile illness and long-term arthralgic sequelae [231]. These viruses replicate in mammalian cells (HEK293) indicating that they have definite control over the host’s UPR system. CHIKV specifically activates the ATF6 and IRE1 cascades and suppresses the PERK pathway [231]. CHIKV NSp4 expression in mammalian cells suppresses eIF2α phosphorylation, which regulates the PERK pathway [231]. The experimental findings showed that SINV induced uncontrolled UPR, which was reflected by the failure to synthesize ER chaperones, followed by increased phosphorylation of eIF2α and activation of CHOP which led to premature cell death [231]. These results highlighted the differences in mechanisms of UPR regulation by similar viruses. Another study showed the activation of XBP1 pathway when neuroblastoma N18 cells were infected with the arboviruses JEV and DENV [232]. This was seen by splicing of XBP1 mRNA and activation of *ERDJ4, EDEM1*, and *p58* genes. Applying small interfering RNA to reduce XBP1 had no effect on cellular susceptibility to the two viruses but enhanced cellular apoptosis [232]. These results suggest that both JEV and DENV trigger the XBP1 signaling pathway and take advantage of this cellular response to alleviate virus-induced cytotoxicity [232].

It is worth mentioning the beneficial role-played by the enzyme activities that are upregulated by UPR signaling. Two studies showed that alpha-glucosidase inhibitors Celgosivir (an iminosugar) and Castanospermine can inhibit DENV production via inhibition of ER-resident alpha-glucosidase in primary human macrophages a Huh-7 and BHK-21 cells, respectively [233,234].

Another study showed that A547 ovarian cancer cells infected with DENV elicited the UPR signaling response [235]. This was confirmed by phosphorylation of eIF2α. It was also shown that different serotypes of DENV activate other UPR pathways such as ATF6 and IRE1. These results showed that different serotypes of DENV have the capacity to modulate different UPR pathways. This unique report showed that different viruses from the same group could activate different UPR pathways [235]. This report also indicated that DENV induces the expression of the protein complex (containing the protein phosphatase 1 and its cofactor GADD34) which leads to enhanced dephosphorylation of eIF2α. Pharmacologically inhibiting GADD34 by Salubrinal, a small molecule inhibitor of this protein complex dephosphorylated eIF2α, dramatically reduced GADD34 and subsequently reduced DENV infection [235].

Thus, specific virus-induced UPR pathway usage depends on the type of viral strain. Even the use of ectopically expressed arbovirus NS proteins alone in mammalian cells could elicit the UPR response. TBEV triggers eIF2α phosphorylation, but the kinase responsible for this process is not yet known. The stress granule component TIA-1 binds TBEV RNA which is recruited to perinuclear sites of viral replication to inhibit viral translation [236]. During TBEV infection in Vero E6 cells, IRE1 and ATF6 pathways are triggered [237]. These pathways contribute to the inhibition of IFN signaling mediated by STAT1 phosphorylation [230]. After VEEV infection of U87MG cells, the UPR PERK arm was activated, and the expression of both ATF4 and CHOP (DDIT3), critical regulators of the pathway, was altered. The expression of the transcription factor early growth response 1 (EGR1) was also induced in a PERK-dependent manner. EGR1 contributed to VEEV-induced cell death by modulating proapoptotic pathways [238]. An overall schematic representation and a summary of findings about UPR and arbovirus replication are summarized in Figure 3 and Table 3.

**Table 3.** Summary of arbovirus and UPR pathway.

Reference	Summary of the study	Conclusions
^[200]^	Studied UPR involvement in WNV infection	WNV activates multiple UPR pathways. XBP1 pathway was not necessary for WNV replication; however, ATF6 was degraded by the proteasome and PERK pathway transiently phosphorylated eIF2α and induced the pro-apoptotic protein CHOP. The host mechanism to counteract WNV infection involved activation of CHOP-dependent cell death.
^[200,229]^		WNV infection activatied UPR via ATF6/IRE1 pathways.
^[229]^		UPR PERK pathway was not activated. The WNV Kunjin strain NS4A and NA4B proteins are potent inducers of UPR. Moreover, sequential removal of hydrophobic domains of NS4A decreased UPR activation. Hydrophobic residues of WNV Kunjin strain NS proteins activate UPR signaling. In contrast, upon infection with WNV, IREI-deficient cells did not illustrate any distinct difference as compared to IREI-positive cells. In the absence of ATF6, other UPR signaling cascades such as PERK and IRE1 pathways could not activate. It was also shown that both ATF6 and IREI are required for STATI phosphorylation, highlighting the necessity of ATF6 for inhibition of innate immune response.
^[232]^	Studied UPR involvement in JEV and DENV infection	JEV and DENV infection activated XBP1 pathway in neuoroblastoma N18 cells. Applying small interfering RNA to reduce XBP1 had no effect on cellular susceptibility to the two viruses but enhanced the cellular apoptosis. Both JEV and DENV trigger the XBP1 signaling pathway.
^[231]^	Studied UPR involvement in CHIKV and SINV infection	CHIKV specifically activates the ATF6 and IRE1 cascades and suppresses the PERK pathway. CHIKV NSp4 expression in mammalian cells suppresses the eIF2α phosphorylation which regulates the PERK pathway. SINV induced uncontrolled UPR, which was reflected by the failure to synthesis of ER chaperones, followed by increased phosphorylation of eIF2α and activation of CHOP
^[235]^	Studied UPR involvement in DENV infection	Showed that A547 ovarian cancer cells infected with DENV elicited the UPR signaling response Different serotypes of DENV have the capacity to modulate different UPR pathways. This unique report showed that the same viruses from the same family could activate different UPR pathways
^[236]^	Studied UPR involvement in TBEV infection	TBEV triggers eIF2α phosphorylation. The stress granule component TIA1 binds TBEV RNA which is recruited to perinuclear sites of viral replication to inhibit viral translation.
^[237]^		During TBEV infection, IRE1 and ATF6 pathways are triggered
^[238]^	Studied UPR involvement in VEEV infection	Following VEEV infection, PERK was activated and the expression of both ATF4 and CHOP (DDIT3) was altered. Expression of EGR1 was also induced in a PERK dependent manner.

CHIKV: Chikungunya virus; DENV: Dengue virus; JEV: Japanese encephalitis virus; RVFV: Rift Valley fever virus; SINV: Sindbis virus; TBEV: Tick-borne encephalitis virus; VEEV: Venezuelan equine encephalitis virus; WNV: West Nile viruss.

## Influenza virus and autophagy

The autophagy pathway is involved in IAV replication [124,239–241]. It was suggested that the induction of autophagosome degradation during IAV infections might restrict virus replication within infected cells [242]. This could be considered an immune mechanism as well, which impairs or restricts IAV replication and virulence [243–246]. IAV infection inhibited autophagy at the stage of autophagosome/lysosome fusion, which led to the accumulation of autophagosomes in A549 cells [124] and evasion of viral antigen presentation [124,247,248].

Bafilomycin A1 (Baf-A1) is a highly specific, but somewhat toxic, inhibitor of vacuolar-type proton (V-H^+^) pumps, which are responsible for the acidification of endosomes and lysosomes, and are necessary for IAV replication [249]. Using Baf-A1 at low sub-toxic concentrations also effectively inhibited IAV replication without impacting host cell viability [240]. Thus, sub-toxic Baf-A1 concentrations can inhibit autophagosomes, either by inhibiting autophagosome formation or by increasing degradative flux [240]. A study on the antiviral activity of statins confirmed that IAV increases autophagosome formation by LC3-II accumulation and inhibits autophagosome maturation/degradation [250]. Law et al. showed induction of functional autophagy by different strains of IAV in primary human blood macrophages, which was detected by the degradation of the autophagy receptor p62 [241]. It was also shown that IAV induced autophagy with no detectable block in the pathway via confirmation of GFP-LC3 puncta and p62 degradation measurements. They also showed that autophagy was not involved in MHC class II-restricted presentation [251]. In a report that studied IAV H3N2 infection, LC3-II began to accumulate a few hours post-infection (hpi) in A549 cells and very early post-infection in Ana-1 macrophages. This also confirmed that H3N2 induced autophagy in both A549 and Ana-1 cells [252].

The IAV M2 and NS1 proteins are associated with autophagy signaling. The NS1 protein binds to p85β, the regulatory subunit of PI3K and activates its signaling pathway, causing the phosphorylation of Akt with subsequent phosphorylation of Beclin-1, which regulates autophagy [253]. M2 keeps autophagy at moderate levels by limiting the degree of lysosome fusion with autophagosomes [124,247]. Gannage *et al*. showed that M2 proton channel activity is not involved in blocking autophagosome degradation. They verified this fact by using amantadine hydrochloride, an inhibitor of M2 ion channel activity, which was unable to abrogate autophagosome accumulation in IAV-infected cells. Finally, they showed that the N-terminal first 60 amino acids of M2 blocks autolysosome formation and acts independently of its proton channel function [124]. However, it was recently shown that the proton channel activity of M2 is involved in this blocking activity [254]. This discrepancy might be related to the strain of the virus used in the study, A/Hong Kong/8/68(H3N2), which was sensitive to amantadine. Fletcher at al. also confirmed that LC3 relocalization during IAV infection depends on the proton channel activity of M2. They showed non-canonical autophagy (independent of ATG genes) was dependent on WD40 CTD of ATG16L1, raising the possibility that activation of the non-canonical autophagy pathway can be triggered by the loss of cellular pH gradients. The cells lacking WD40 CTD were unable to support LC3 lipidation [255]. Su et al. found that M2 ubiquitination was crucial for infectious virus particle production, but not required for blocking late-stage autophagy. The levels of LC3-II at 6 hpi were threefold higher in M2-K78R mutant virus-infected cells than in WT-infected cells. Thus, a M2 ubiquitination-defective mutation (M2-K78R) induced autophagy even earlier than wild-type (WT) virus [256].

The mTOR signaling pathway negatively regulates autophagy [257,258]. The phosphorylated form of the effector protein kinase mTOR is known to inhibit autophagy [259]. It has been proposed that the highly pathogenic avian influenza virus, H5N1, induces autophagy in MEF cells by suppressing phosphorylated mTOR signaling. Thus, inhibition of autophagy could reduce H5N1-mediated cellular damage [260]. However, autophagy in human epithelial cells involved AKT, a tumor suppressor protein TSC2 and mTOR. Sun and colleagues also showed that H5N1 HA was responsible for stimulating autophagy [261]. A study demonstrated that the activation of the PI3K/AKT pathway, which is closely related to the autophagic process, has a biphasic effect on IAV replication [262]. In this regard, Datan et al. showed that lethal autophagy and protective autophagy are activated in different ways. In lethal autophagy, mTORC2 upregulates p70S6K activity, that is required for LC3-II formation which interestingly increases viral production by delayed lysosome activity. Blocking PI3K, mTORC2 or p70S6K activity prevented lethal autophagy and limited infectious virus production [263]. Deficiency in autophagy caused impaired survival of memory CD8^+^ T cells during infection with IAV [264].

**Figure 4.**
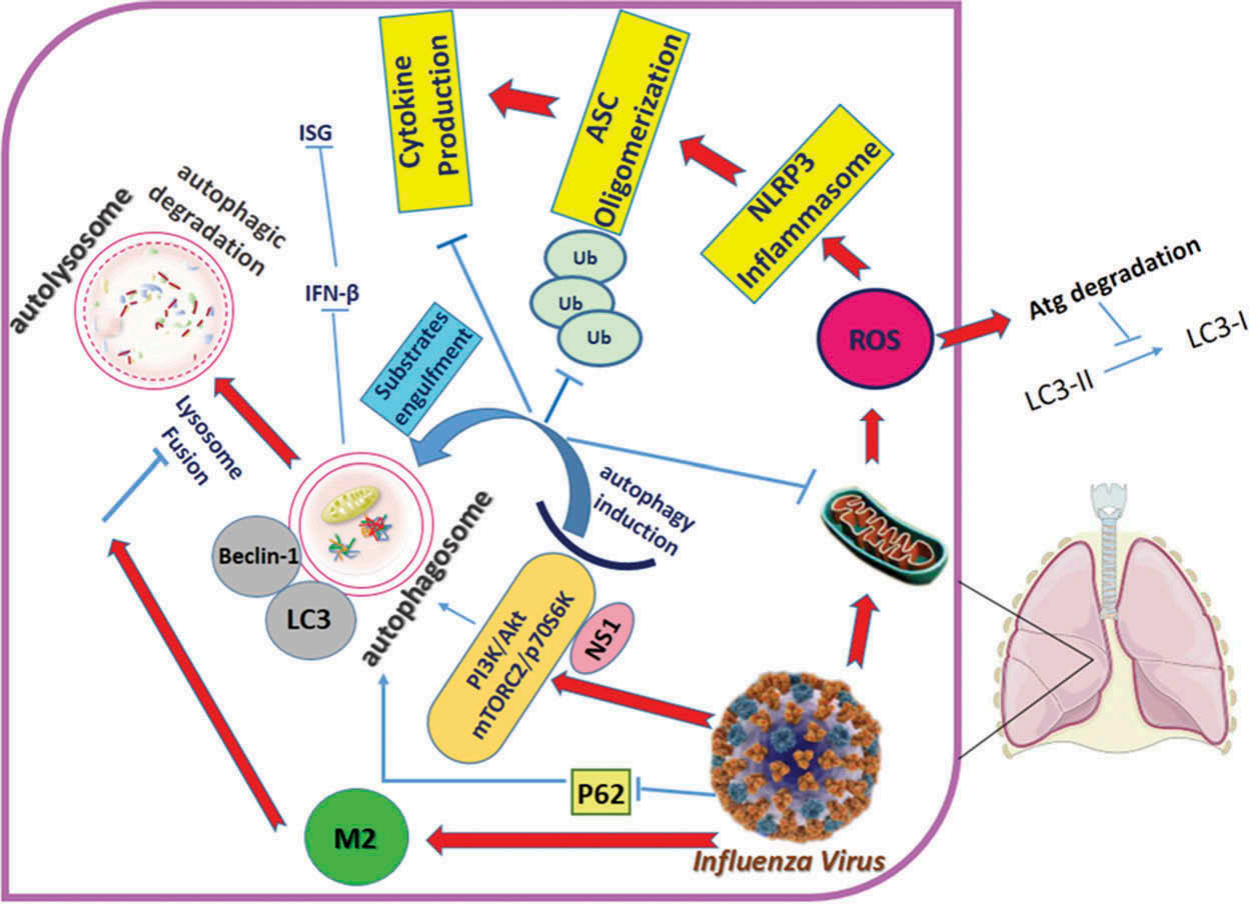
Autophagy Signaling During Influenza A Virus Infection. Influenza A virus (IAV) induces the NLRP3 inflammasome, which causes mitochondrial damage and release of ROS, which prevents the conversion of LC3-II to LC3-I by degrading Atg4 and leads to increased levels of LC3-II. NLRP3 forms an inflammasome complex with ASC and induces the production of inflammatory cytokines. IAV also binds to Beclin1 by the viral M2 protein. It up-regulates the expression of several autophagy-related genes, which can increase autophagic flux. M2 also contains an LC3-interacting region (LIR) which is required for influenza virus subversion of autophagy; this leads to LC3 redistribution to the plasma membrane in infected cells. The complex P-mTORC2/p70S6K blocks lethal autophagy. Autophagosome formation blocks IFN-β and reduces ISG expression [modified from [337]].

Optimal cytokine levels exert protective effects against IAV replication [265,266]. Autophagy is a key mechanism contributing to the inflammatory responses induced by IAV infections [241]. Pan and colleagues reported that after H5N1 infection, NF-_k_B signaling-induced autophagosome formation was activated in both human lung epithelial cell lines and in mouse lung tissues [264]. The positive feedback between autophagy and NF-_k_B and p38 MAPK signaling cascades could be an important mechanism contributing to H5N1-induced lung inflammation [264]. Ectopic P-granule autophagy protein five homolog (EPG5), which is essential for basal autophagy and ATG gene complex functions in the formation of degradative autolysosomes, regulates basal expression of multiple cytokines in the lung [266]. It was also reported that induction of autophagy by IAV infection reduces interferon-stimulated gene (ISG) expression in infected cells by limiting IFN-β expression, which may benefit viral replication and spread (Figure 4) [267].

The molecular mechanisms underlying the reduction of major cellular antioxidant enzyme group superoxide dismutase 1 (SOD1) during IAV infection, which results in over-production of reactive oxygen species (ROS), clearly involves autophagy. Silencing the LC3 gene in A549 cells, which normally supports the critical role of autophagy in ROS, increases in the early phases of IAV infection [268]. IAV also induces the nucleotide-binding domain and leucine-rich repeat (NLR) family, including the pyrin domain containing 3 (NLRP3) inflammasome, causing mitochondrial damage and release of ROS. NLRP3 forms an inflammasome complex with ASC (essential adaptor of inflammasomes) and caspase-1, thus inducing the production of IL-1α and IL-18 in response to mitochondrial ROS [269]. Upon infection with IAV, the cytosolic PRR Nod2 and its downstream regulator RIPK2 induce mitophagy by inducing phosphorylation of ULK1 to prevent excessive activation of the NLRP3 inflammasome (Figure 4) [270].

In association with efforts to develop IAV treatment strategies, a study evaluated the role of autophagy induction using a Beclin-1 expression plasmid before and after IAV inoculation. This therapeutic approach of autophagy induction inhibited virus replication at 24 and 48 hpi, but the prophylactic approach was not successful [271]. Another study identified a role for K27-linked ubiquitination in tripartite motif (TRIM)23 GTPase function and its ability to activate TANK-binding kinase 1 (TBK1)-mediated autophagy, which together are key components of selective autophagy for different viruses including IAV. This could be a basis for therapeutics against diseases caused by dysregulation of autophagy such as IAV [272]. Scientists have recently designed a peptide called Kα2-helix, derived from the viral FLICE-like inhibitor protein (vFLIP) of Kaposi’s sarcoma-associated herpesvirus (KSHV). They detected the autophagy activity of this compound at the initial stages of IAV infection which inhibited the binding of FLIP to the E2-like enzyme Atg3 without affecting the interaction of LC3 and Atg3. Then, they fused it with a protein transduction domain [YGRKKRRQRRR] of the HIV-1 TAT protein. This construct showed significant inhibition of lethal doses of H5N1 or H1N1 virus replication and transmission by destabilizing the viral membrane. Therefore, these artificial constructs may represent promising antiviral agents to control various IAV subtypes [273]. Fluorescent labeling of individual viral particles has been used for decades [274], but was recently adapted to reveal autophagic trafficking of IAV H9N2 independent of Rab5, which could provide a better understanding of the fundamental relationship between autophagy and virus entry [275]. The interaction of IAV with the host autophagy pathway is illustrated in Figure 4 and a summary of autophagy and influenza virus infection is summarized in Table 4.

**Table 4.** Summary of influenza virus infection and autophagy pathway.

Reference	Summary of the study	Conclusions
^[124,247]^	IAV infection in A549 cells and inhibition of autophagosome/lysosome fusion	Evade of viral antigens presentation by M2 protein independent of M2 proton ion channel function
^[240,250]^	Administration of statins and Baf-A1 at low concentration on IAV-infected cells	Confirmed IAV increases autophagosome formation by LC3-II accumulation and inhibits autophagosome maturation/degradation
^[241,251]^	Induction of functional autophagy by different IV strains in primary human blood macrophage	Induction of functional autophagy by degradation of the autophagy receptor p62
^[252]^	H3N2 infection in both A549 and Ana-1 cells	LC3-II accumulation and autophagy induction a few hpi in both A549 and Ana-1 cells
^[254]^	IAV (A/Hong Kong/8/68(H3N2)) infection (the strain sensitive to amantadine)	Blocking autophagy occurred by proton channel activity of M2
^[255]^	LC3 relocalisation during IAV infection	Blocking autophagy by proton channel activity of M2, dependency on WD40 CTD of ATG16L1 in non-canonical autophagy pathway
^[256]^	M2 ubiquitination-defective mutation (M2-K78R)	Ubiquitination of M2 is not required for blocking late-stage autophagy
^[259]^	Studied on effector protein kinase; mTOR	Phosphorylated form of mTOR inhibits autophagy
^[260]^	H5N1 infection in mouse embryonic fibroblast (MEF) cells	H5N1 induced autophagy by suppressing phosphorylated mTOR signaling
^[261]^	H5N1 infection in human epithelial cells	H5N1 HA glycoprotein was responsible for autophagy induction involving AKT, TSC2 and mTOR
^[263]^	Studied lethal autophagy during IAV infection	mTORC2 upregulates p70S6K activity, increases LC3-II formation and viral production by delayed lysosome activity
^[264]^	H5N1 infection in human lung epithelial cell lines and mouse lung tissues	The positive feedback between autophagy and NF-_k_B and p38 MAPK signaling cascades could be an important mechanism contributing to H5N1 lung inflammation
^[267]^	Studied ISG expression in IV-infected cells	Induction of autophagy by IAV infection reduces ISG expression by limiting IFN-β expression, which may benefit viral replication
^[268]^	Silencing LC3 gene in A549 cells and molecular mechanism underlying the reduction of SOD1 during influenza infection	Supported the critical role of autophagy in the ROS increase in the early phase of flu infection
^[269]^	IAV infection and mitochondrial damage	Induction of the NLRP3 inflammasome causing mitochondrial damage and release of ROS and production of IL-1α and IL-18
^[271]^	Two cell lines MDCK and MDCK-SIAT1 were transfected with Beclin-1 expression plasmid before and after IV inoculation	The therapeutic approach of autophagy induction inhibited the virus replication at 24 and 48 hr post-infection
^[272]^	Studied the role of K27-linked ubiquitination in TRIM23 GTPase function and its ability to activate TBK1-mediated autophagy	Together are key component of selective autophagy for IAV infection. A basis for therapeutics against IAV caused by dysregulation of autophagy
^[273]^	designed Kα2-helix peptide derived from vFLIP of Kaposi’s sarcoma-associated herpesvirus (KSHV), Then fused it with the TAT peptide of HIV-1	Autophagy activity at the initial stages of IAV infection which inhibited the binding of FLIP to the E2-like enzyme Atg3 without affecting the interaction of LC3 and Atg3 significant inhibition on lethal doses of H5N1 or H1N1 replication and transmission by destabilizing the viral membrane
^[275]^	QD-based SVT technique combined with multi-colour visualization of the transport process of individual viruses	Provided a better understanding of the fundamental relationship between autophagy and virus entry

## Influenza virus and apoptosis

**Figure 5.**
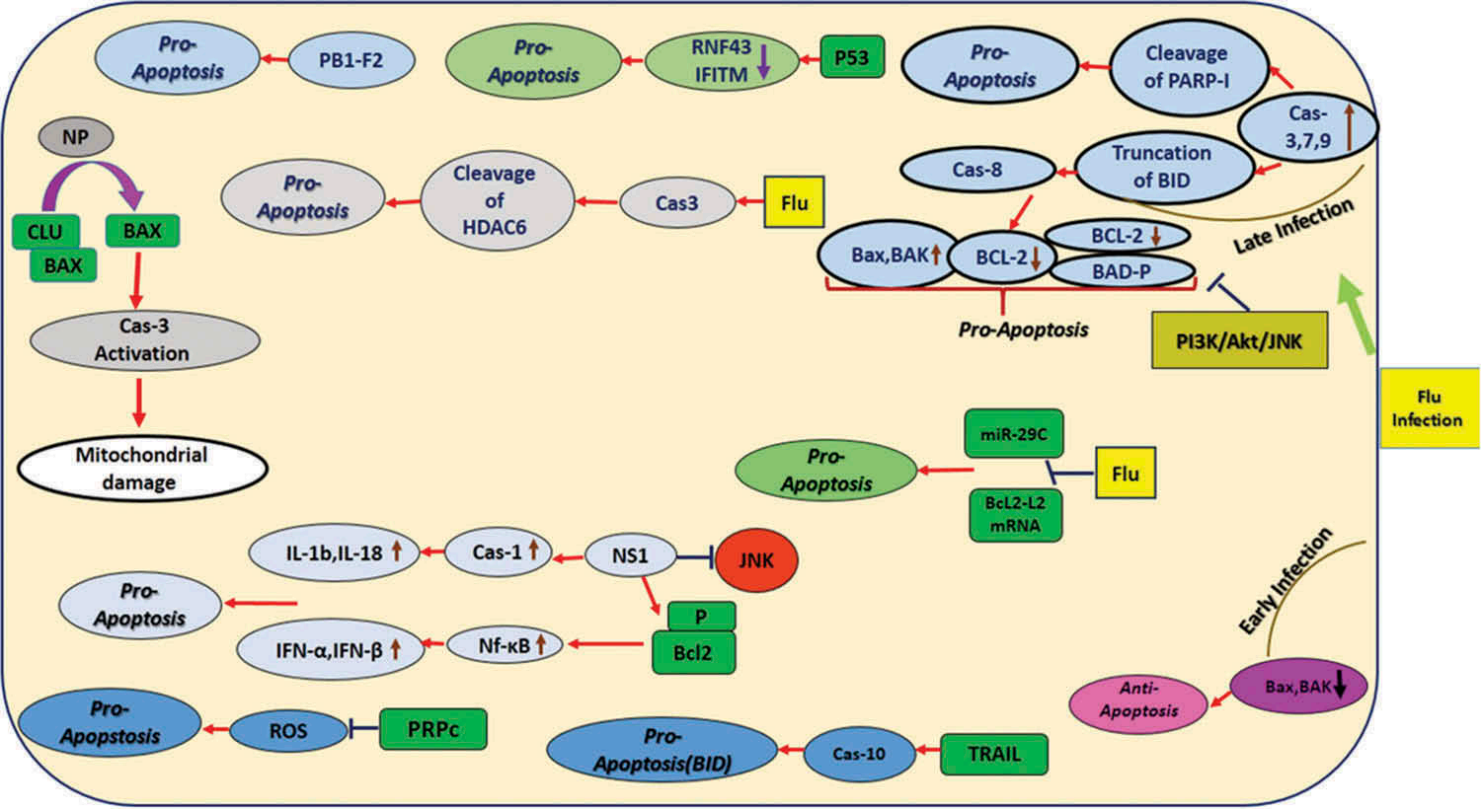
Apoptosis Signaling During Influenza A Virus Infection. IAV infection affects apoptosis in early and late infection. In early infection, it has anti-apoptotic effects by decreasing Bax and BAK, but in late infection, it is pro-apoptotic by increasing Bax and BAK, cleavage of PARP-I, truncation of the BID, phosphorylation of BAD and decreasing Bcl-2. Cellular factors P53, miR-29C, TRAIL, CLU and PRPc are involved in IAV infection through the apoptosis pathway. IAV proteins PB1-F2, NP, and NS1 are also involved.

Several studies have highlighted the importance of apoptosis induced by IAV in different models. These include using A/PR/8/34 H1N1 [276], A/Wenshan/01/2009 H1N1 [277], or A/New York/55/2004 (H3N2) (NY55), A/Puerto Rico/8/1934 (H1N1) (PR8), and 2009 pandemic swine origin influenza virus (SOIV) (A/California/07/2009) [278]. Apoptosis and autophagy in IAV infection are interconnected [124,239,261,278]. Zhirnov and Klenk reported delayed apoptosis and highly stimulated autophagy in IAV-infected cells [125]. Yeganeh and colleagues also highlighted that autophagy activation occurred 8–14 hr earlier than apoptosis [279]. PR8-induced caspase-7,-3,-9 activation in MEF cells coincided with both the cleavage of PARP-1, a distal event in apoptosis signaling, as well as the truncation of BID, a specific substrate of caspase-8 in the extrinsic apoptotic signaling pathway. The alteration of Bax and Bak levels caused an increase in the ratio of pro-apoptotic (Bax and Bak) proteins to anti-apoptotic (Bcl-2) protein (Figure 5) [279].

Several studies have investigated the role of pro- and anti-apoptotic Bcl-2 family members including Bcl-2, Bax, and Bak in IAV infection [280–282]. In silico target prediction analysis revealed complementarity of microRNA miR-29c to the 3‘ untranslated region (UTR) of BCL2L2 mRNA. It is involved in apoptosis induced by IAV infection. IAV infection affects miR-29c and down-regulates Bcl2-L2 expression, which leads to apoptosis promotion in A549 cells (Figure 5) [283]. Mclean and colleagues showed that pro-apoptotic protein Bak has antiviral effects and its expression is significantly down-regulated during IAV infection. Bak could suppress IAV replication in MDCK cells at 24 hpi. But Bax was activated at 24 hpi. Lack of Bax prevented influenza A virus-induced apoptosis and caused diminished viral replication [280]. However, Yeganeh et al., showed that Bax and Bak are highly expressed in MEF cells at 18 hpi, but the abundance of both proteins was reduced at 24 hpi [279]. This contradiction between the results of these two studies might be related to the time points sampled and the different cell lines used.

The IAV NS1 protein inhibits the JNK pathway in MDCK cells [284]. However, Bax expression, which affects IAV infection, is negatively regulated by the PI3K/Akt/JNK pathway in IAV-infected cells. This leads to JNK-dependent inhibition of Bax-mediated apoptosis in A549 cells [285]. In contrast to the Bak and Bax antiviral effects, PR8 infection in A549 cells induced phosphorylation of another pro-apoptotic Bcl-2 family member, BAD, at residues S112 and S136 in a temporal manner and virus-induced cytopathology and cell death were considerably inhibited in BAD-deficient cells [278]. Chung et al. highlighted in a transcriptomic study that apoptosis marker genes were expressed early in infection, at 8 hpi, which is a host strategy to limit viral infection. The PB1-F2 viral gene was suggested to be responsible for this induction [286]. It has been suggested that the influenza virus could facilitate the progress of its pathogenesis and transmission by induction of apoptosis in neutrophils [287] and NK cells [288].

Different IAV-induced caspase activities also were studied. Stasakova et al. showed the role of IAV NS1 protein to provoke caspase-1 activation in primary human macrophages, resulting in fast apoptosis and release of high levels of interleukins 1b and 18. These results were confirmed by a PKR inhibitor [289]. Influenza virus NS1 protein interacts with β-tubulin, which arrests the infected cells in G2/M phase and affects Bcl-2 phosphorylation [290,291]. This, in turn, activates NF-_k_B and induces INF-α and INF-β [292], which then induce the apoptosis cascade by induction of SOCS-3 expression [293,294]. Shiozaki et al., showed the cellular pro-apoptotic protein Siva-1 in A549 cells was involved in IAV replication through caspase activation [295]. Another study showed that apoptosis induction in IAV-infected A549 cells correlates with NP expression through the intrinsic pathway. They used the A/California/07/2009(H1N1) strain, which lacks a functional PB1-F2 protein (proapoptotic factor). Clusterin, a host protein, attenuates IAV NP-induced apoptosis and IAV replication [296]. Yang et al. demonstrated that 2009 pandemic H1N1 A/Beijing/501/2009 could induce caspase-3-dependent apoptosis in the A549 cell line [292]. Husain and Harrod showed that caspase-3-mediated histone deacetylase 6 (HDAC6) cleavage in IAV-infected MDCK cells caused further damage in these cells [297]. In general, caspase-3 activity and DNA fragmentation serve as indicators of early and late apoptosis, respectively.

Studies also have used targeted mutagenesis of viral and host proteins to further assess the role of apoptosis in IAV infection. Su et al. demonstrated that M2-K78R mutant viruses (a ubiquitination-defective mutation in viral M2) induced apoptosis in HEK293T cells more than wild-type (WT) viruses. They concluded that this mutation enhances early and late apoptosis which led to more cell death [256]. In another study, fucosyltransferase enzymatic activity was evaluated in effector and memory CD4 + T cells. This enzyme mediates the addition of the terminal fucose of sLex to PSGL-1 on CD4 + T cells, which is required for the generation of functional memory CD4 + T cells. Greater cell death was observed in Fut4/7^−/-^ mutant than in WT cells at 8 dpi by measuring the levels of cleaved caspase-3, together with propidium iodide staining. These data highlight that expression of fucosyltransferase genes in CD4+ effector responses to IAV infection plays a role in maintaining their survival [298]. In addition, another group showed that PrP^C^-null mice (Prnp°^/0^) were more highly susceptible to IAV than were WT mice [299]. PrP^C^ is the cellular membrane normal form of the PrP^Sc^ prion glycoprotein. Prnp°^/0^ mice showed higher apoptosis levels through activated caspase-3. They suggested PrP^C^ might exert its protective anti-apoptotic activity by disturbing oxidative stress-induced apoptosis, and not by directly affecting viral replication in lungs [299].

The apoptosis-inducing activity of IAV A/Chicken/Hubei/489/2004 (H5N1) was studied in MDCK, Vero and HeLa cells. Infected MDCK cells, but not Vero and HeLa cells, showed caspase-8-dependent apoptosis. Thus, it was suggested that this virus-induced apoptosis in a cell-specific manner [300]. Avian H5N1 promotes TNF-related apoptosis-inducing ligand (TRAIL) in human monocyte-derived macrophages (MDMs). Apoptosis was induced by TRAIL-activated caspase-10, resulting in the activation of BID and the intrinsic apoptosis pathway (Figure 5) [301].

The polymerase acidic protein of influenza virus (PA)-X, encoded by the PA mRNA as an IAV virulence modulation factor, showed loss of expression in pH1N1 and H5N1 viruses in A549 cells, which was associated with increased virulence and apoptosis and host inflammatory response in mice [302]. Another group showed nucleolin (NCL), a novel PA-interacting host protein, significantly decreased IAV replication in the case of H5N1 infection in HEK293T cells by decreasing inflammatory responses. The antiviral mechanisms of NCL may pave the way to develop new anti-influenza drugs [303].

A study conducted by Stawowczyk and colleagues showed that duck AvIFIT protein might be related to the induction of cell apoptosis, similar to human IFIT2 [304]. Another study found duck AvIFIT protein played a critical role in host immune response to H5N1. This was mediated by AvIFIT binding influenza NP protein, which increased the expression of IRF1, IRF7, IFNa, IFNb, TNFa, NF-_k_B and IL-12, which in turn magnified IFN signaling and arrested cell growth [305]. IL-36γ, a key mediator of immune protection during IAV infection, promoted lung alveolar macrophage survival by decreasing apoptosis and limiting viral replication [306].

The study conducted by Lee et al. confirmed the onset of apoptosis at early phases of infection. They also showed the switch from apoptosis to pyroptosis (cell death pathway) in normal or precancerous human bronchial epithelial cells under the regulation of type I IFN signaling to initiate pro-inflammatory responses against IAV infection [307]. High antibody response to influenza vaccine showed decrease in post-vaccination tumor necrosis factor-like weak inducer of apoptosis (TWEAK) levels, which is a pro-inflammatory molecule primarily produced by leukocytes [308]. Infection of alveolar type II epithelial cells with IAV along with antioxidant treatment could decrease influenza virus replication and apoptosis induction [309].

To screen some host proteins required for secondary bacterial infection after H9N2 virus infection, Ma et al. used iTRAQ proteomics coupled with nano-LC–MS/MS technology. They noticed the differentially higher expression of proteins in host cellular pathways including apoptosis, such as apoptotic protease-activating factor 1 [310]. Other proteomic screens of IAV-infected A549 [311–313] and of primary human bronchial airway cells [314,315] also highlighted dysregulated apoptotic (and other) proteins.

Additional studies examined the role of P53 in influenza infection, and apoptosis induction via attenuation of host ubiquitin ligase RING finger protein 43 (RNF43) [316]. IFITM proteins also were inhibited [317], which could be attractive antiviral targets. The molecular mechanisms of selenium nanoparticles, modified on their surfaces by amantadine (Se@AM), and their effects on IAV infection, demonstrated caspase-3 inhibition and a decrease in the level of ROS to trigger AKT pathways. These are additional promising antiviral targets against H1N1 IAV [317]. The interaction of influenza A virus with the host apoptosis pathway is illustrated in Figure 5 and a summary of apoptosis and influenza virus infection is summarized in Table 5.

**Table 5.** Summary of influenza virus infection and apoptosis pathway.

Reference	Summary of the study	Conclusions
^[279]^	Used A549 cells and mouse embryonic fibroblasts to study the cross-talk between autophagy and apoptosis in PR8 infection	PR8 infection simultaneously induced autophagy and apoptosis. Cleavage of PARP-1 and truncation of BID increased the ratio of pro-apoptotic protein to anti-apoptotic protein
^[283]^	In silico target prediction analysis of complementarity of miR-29c to the 3‘ UTR of BCL2-L2 mRNA in A549 cells	IAV infection affected miR-29c and down-regulated Bcl2-L2 expression, which led to apoptosis promotion
^[280]^	Studied IAV infection in mouse embryonic fibroblasts which lack Bax and/or Bak but express functional Bcl-2	Bax induced apoptosis and virus replication, while Bak suppressed apoptosis and viral replication at 24 hpi
^[285]^	Studied to define how PI3K/Akt/JNK pathway regulates IV-induced apoptosis.	Bax expression is negatively regulated by the PI3K/Akt/JNK pathway in IAV-infected cells thus inhibit JNK-dependent, Bax-mediated apoptosis
^[278]^	PR8 infection induced phosphorylation of BAD	Virus-induced cytopathology and cell death were considerably inhibited in BAD-deficient cells
^[286]^	Transcriptome study on apoptosis marker genes in IAV infection	PB1-F2 viral gene was found to be responsible for apoptosis induction in early infection stage
^[288,338]^	Studied progress of IAV pathogenesis and transmission	Induction of apoptosis in neutrophils and NK cells
^[289]^^[290]^^[284]^^[292]–294^^[295]^	-Studied influenza virus NS1 protein	-Caspase-1 activation, fast apoptosis and release of IL-1b and IL-18 -Interaction with tubulin polymerization, arrest of the infected cells in G2/M phase and effect on Bcl-2 phosphorylation -Inhibition of JNK pathway -Activation NF-_k_B and induction of INF-α and INF-β, induction of SOCS-3 expression and induction of apoptosis -Siva-1 involvement for IAV replication through caspase activation
^[296]^	Studied influenza virus (A/California/2009(H1N1) strain) NP protein in A549 cells	Apoptosis induction correlated with NP expression through intrinsic pathway
^[292]^^[297]^^[256]^^[298]^^[299]^ ^[317]^	Studied caspase-3 in IAV infection	−2009 pandemic H1N1 A/Beijing/501/2009 induced caspase-3-dependent apoptosis in cell line A549 -Caspase-3-mediated HDAC6 cleavage in IAV-infected MDCK cells caused further damage to the cells -M2-K78R mutant viruses induced apoptosis more than WT viruses -Fucosyltransferase genes in CD4+ effector responses to IAV infection plays a role in maintaining their survival -Prnp0/0 mice showed higher apoptosis level through activated caspase-3 -Se@AM demonstrated caspase-3 activity inhibition and decreasing the level of ROS to trigger AKT pathways
^[300]^	Studied caspase-8 in IAV infection	AIVHubei489 (H5N1) showed caspase-8-dependent apoptosis in MDCK
^[301]^	Studied caspase-10 in IAV infection in human monocyte-derived macrophages	H5N1 promoted TRAIL and activated caspase-10-depndent apoptosis through activation of BID and intrinsic pathway
^[302]^^[303]^^[304,305]^ ^[316,317]^	Anti-influenza drugs targets	-(PA)-X, a virulence modulation factor of IAV showed loss of expression and induced apoptosis in A549 cells -Nucleolin (NCL), a novel PA-interacting host protein decreased IAV replication and inflammatory responses -Duck AvIFIT protein, similar to human IFIT2 induces apoptosis by binding influenza NP protein and enhancing the IFN signaling -Attenuation of host ubiquitin ligase RNF43 by P53 and inhibiting IFITM proteins
^[307]^	Studied apoptosis in respiratory epithelial cells and PL16T human precancerous respiratory epithelial cells	Onset of apoptosis at early phases of infection and switch from apoptosis to pyroptosis in normal and precancerous cells under type I IFN signaling

## Influenza virus and UPR

**Figure 6.**
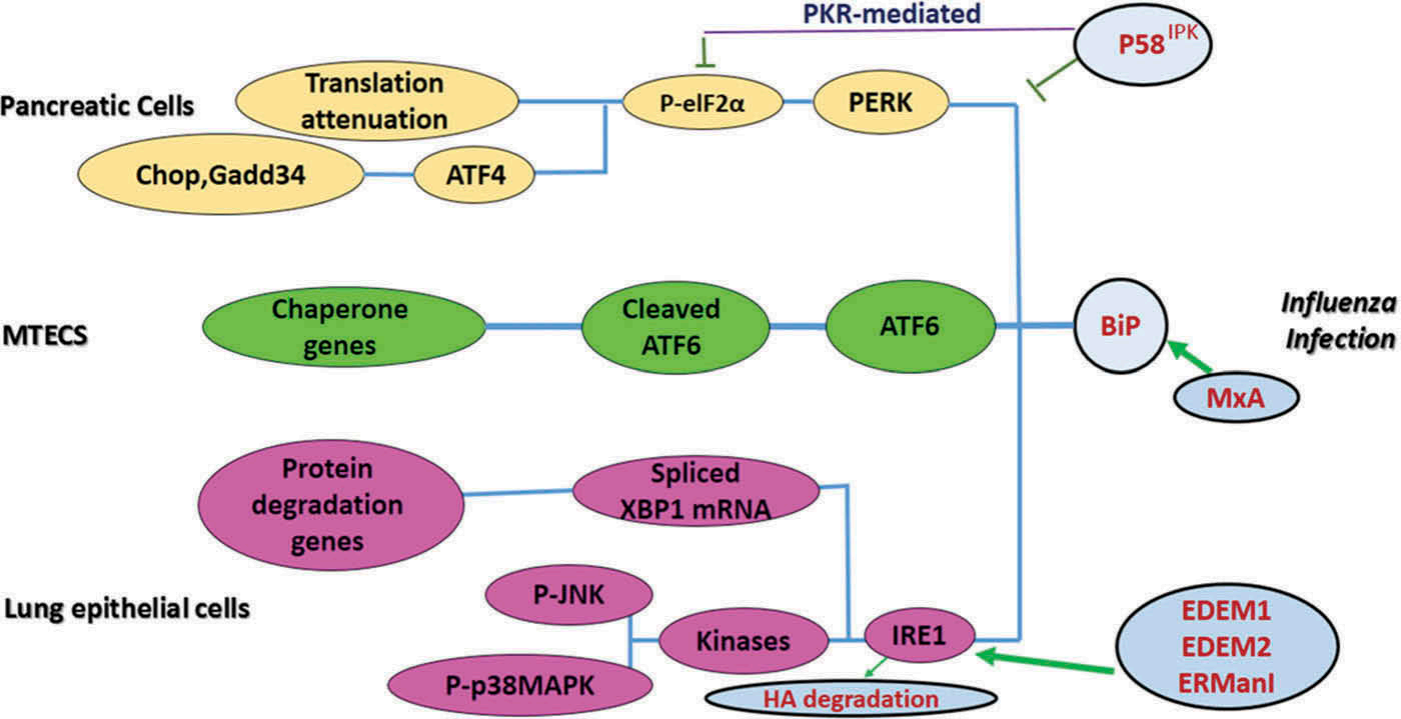
UPR Signaling During Influenza A Virus Infection. Upon IAV infection, BiP is released from UPR proteins (e.g. PERK, ATF6, and IRE1) and facilitates their activation. IRE1 activation results in the splicing of XBP-1 mRNA in the cytoplasm, leading to its nuclear translocation and transcription of UPR target genes. It mediates HA degradation by involvement of three class I α-mannosidases EDEM1, EDEM2, and ERManI. Upon activation of PERK, eIF2a is phosphorylated and blocks protein synthesis. IAV targets eIF2α by inducing P58IPK, then regulates its mRNA translation by PKR-mediated and PERK-dependent mechanisms. ATF6 translocates to the Golgi apparatus where it is cleaved, then moves to the nucleus and targets ER chaperone genes. IAV targets this pathway using MxA.

Hassan et al. showed for the first time the role of ER stress and UPR in the pathogenesis of IAV infection in lung epithelial cells. They reported that IAV infection activates the IRE1 pathway with subsequent XBP-1 mRNA splicing, with little or no effect on the PERK and ATF6 pathways [318]. It was also shown that human myxovirus resistance gene A (MxA) was responsible for the antiviral activity against IAV. MxA is involved in ER stress-induced events, such as BiP mRNA expression and XBP-1 mRNA processing in IAV-infected cells (Figure 6) [319]. Another study reported that IAV infection induces ER-stress via ATF6 and ERp57, but not CHOP. This study was conducted in murine primary tracheal epithelial cells (MTECS), one of the primary sites of IAV infection. They also reported that ER stress in infected cells was related to apoptosis through caspase-12 which plays a central role in the initiation of ER stress-induced cell death [320].

P58^IPK^ is an Hsp40 family member and interferon-induced kinase. After IAV infection in NIH 3T3 cells and ER stress, the posttranscriptional activation of P58^IPK^ occurs which may act synergistically with UPR-mediated transcriptional activation to help influenza virus escape from both PKR- and PERK-mediated translational repression [321]. Another study showed that P58^IPK^ regulates the eukaryotic translation initiation factor eIF2a, and then regulates IAV mRNA translation and infection through a PKR-mediated mechanism, which is independent of PERK [321,322].

Recently, scientists showed that influenza virus HA glycoprotein induced a strong innate antiviral response through activating ER stress via ER-associated protein degradation (ERAD) which mediates HA degradation. They suggested that three class I α-mannosidases were distinguished to play a critical role in initiating HA degradation, including EDEM1, EDEM2, and ERManI (Figure 6). This was confirmed by silencing these genes, which increased HA expression [323]. This outcome confirmed the previous study’s results of ER-stress activation by IAV infection through the IRE1 pathway [318].

The interaction of influenza A virus with the host UPR pathway is illustrated in Figure 6 and a summary of UPR and influenza virus infection is summarized in Table 6.

**Table 6.** Summary of influenza virus infection and UPR pathway.

Reference	Summary of the study	Conclusions
^[318]^	Studied ER stress and UPR in IAV infection in the lung epithelial cells	IAV infection activates IRE1 pathway with subsequent XBP-1 splicing
^[319]^	MxA antiviral activity against IAV	Involved in BiP mRNA expression and XBP-1 mRNA processing
^[320]^	Studied UPR in murine primary tracheal epithelial cells	IAV infection induced ER-stress via ATF6 and ERp57, but not CHOP, through caspase-12
^[321]^	P58^IPK^ role in IAV infection	-May act synergistically with the UPR-mediated transcriptional activation to help IV relief from both PKR- and PERK-mediated translational repression
^[322]^		-Regulates eIF2a, and then regulates IV infection independent of PERK
^[323]^	Studied HA glycoprotein degradation	Innate immune induction by HA through ERAD by the critical role of class I α-mannosidases (EDEM1, EDEM2, and ERManI) in initiating HA degradation

## HIV and autophagy

The aim of autophagy is to reestablish homeostasis in response to a variety of stress conditions. HIV infection represents one of the best-characterized systems in which autophagy is disarmed by a virus using multiple strategies to prevent the sequestration and degradation of its proteins and to establish chronic infection [324]. HIV infection has been shown to induce and inhibit autophagy. The differences in the data are almost certainly due to differences between the cell types used [325].

In contrast to CD4 + T-cells, macrophages are not depleted during HIV infection, but are considered a reservoir for the virus. Autophagy plays a double role in infected macrophages. HIV infection results in autophagy induction in the early steps of viral infection and blocks autophagy to avoid the elimination of the virus. HIV Tat causes impairment of autophagy in macrophages. Tat inhibits autophagy in neurons and also inhibits autophagosome/lysosome fusion by interaction with LAMP2A; this might be the reason that neurons will proceed to apoptosis because of inhibition of autophagy in HIV-associated neurodegenerative disease (HAND). Tat induces autophagy by interaction with BAG3 in glial cells [324]. Nef, an HIV-1 accessory protein, interacts with IRGM and then IRGM is capable of activating autophagy by favoring the assembly of a ULK1/BECLIN-1/ATG16 complex. Although autophagy levels are upregulated during infection, both antiviral and immune properties of this process are severely inhibited by HIV infection. An important example is the interaction of Nef and Beclin-1 which inhibits autophagosome maturation [324].

HIV ENV has different effects in different type of cells. The HIV envelope glycoprotein GP120 is released from infected glial cells and able to induce autophagy in neurons. In dendritic cells (DCs), HIV ENV inhibits the autophagic process through activation of mTOR and S6K. This event leads to the inhibition of viral degradation and accumulation of viral particles in DCs. Moreover, autophagy inhibition by LC3 and ATG5 silencing increases HIV transfer to CD4 + T-cells. Autophagy in HAND has also been evaluated. A contribution of autophagy alteration to HAND pathogenesis has been proposed based on the evidence of abnormal accumulation of large autophagosomes and an increase in autophagic markers like ATG5, ATG7, and LC3II in post-mortem brains of HIV patients with encephalitis [324].

## HIV and apoptosis

Patients infected with HIV experience a progressive decline in the number of CD4 + T-cells, which results in immunodeficiency and susceptibility to opportunistic pathogens and malignancies. The primary basis of a decline in the number of T-cells is an increase in apoptosis of CD4 and CD8 T-cells. Although immune system activation due to viral infection can be the cause of apoptosis in these immune cells, analyses showed that HIV specific proteins have a role in apoptosis induction [326]. The HIV-1 envelope protein GP120/GP41 is associated with apoptosis in infected and uninfected CD4 T-cell lymphocytes [327]. Since viruses are intracellular parasites that use the host cell machinery to replicate and get assembled, many viruses have mechanisms to prevent apoptosis for the benefit of the virus itself, but such anti-apoptotic machinery is not present in HIV. HIV-encoded proteins can induce apoptosis in infected and in uninfected cells (i.e. paracrine death) [326]. In HIV infection, the virus binds to CD4 + T-cells by binding to the CD4 receptor. The virus is subsequently internalized into the T-cell where HIV Tat protein is thought to increase the expression of Fas receptor, resulting in excessive apoptosis of CD4 + T-cells by the extrinsic pathway [328].

**Figure 7.**
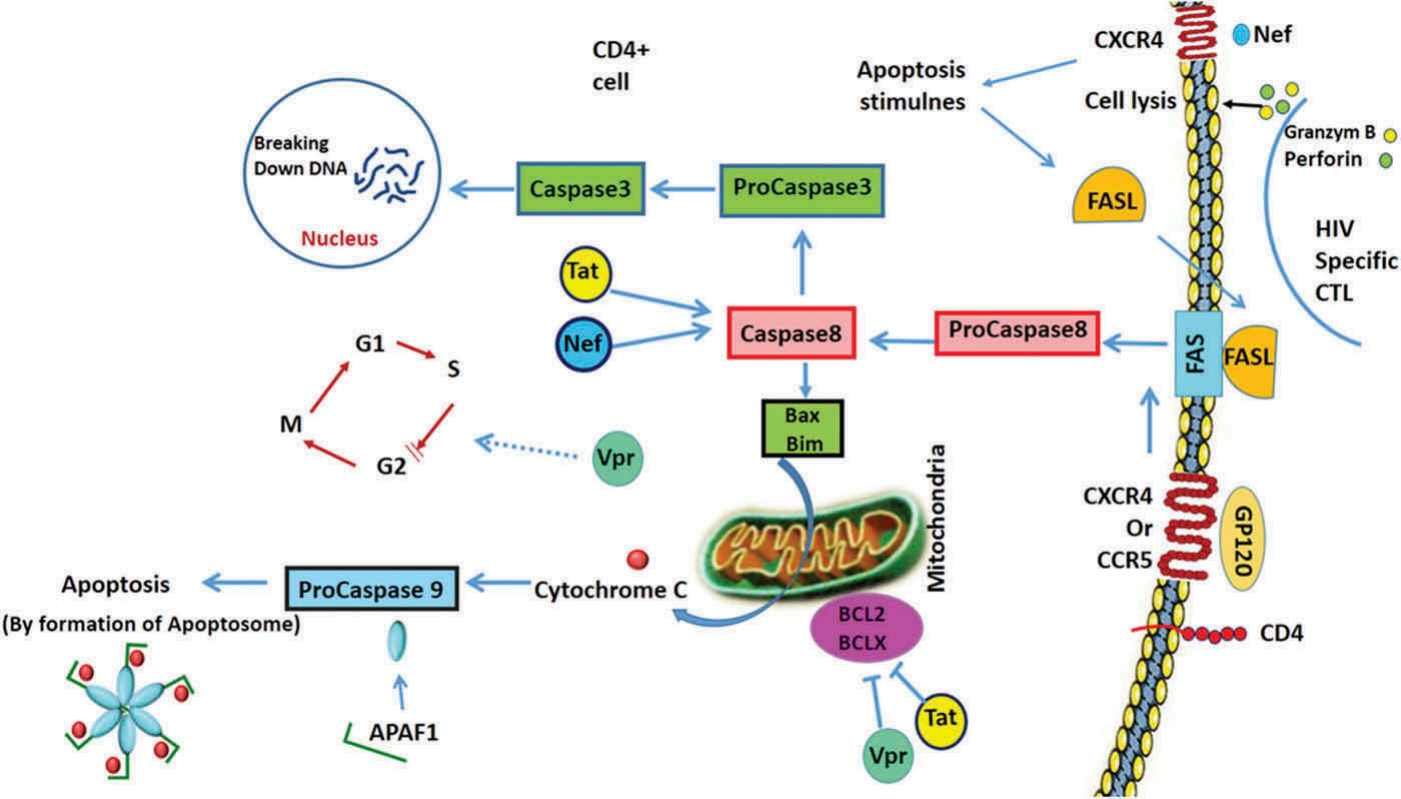
Apoptosis Signalling in T-Helper Cells During HIV Infection. HIV proteins are involved in apoptosis. GP120 attachment to CD4 receptor and CCR5 or CXCR4 can induce the extrinsic pathway in a Fas-dependent manner. GP120 induces Bax expression which activates the intrinsic pathway of apoptosis by the release of cytochrome C (Cyto c) and formation of the apoptosome. Vpr causes cell cycle arrest at the G2 stage. Tat and Nef activate the expression of caspase 8 which changes procaspase 3 to caspase 3 and results in DNA degradation. Tat and Vpr down regulate Bcl2 and BclXL.

GP120 is an HIV viral envelope glycoprotein that can bind to and cross-link the CD4 receptor and the chemokine co-receptors (CCR5 and CXCR4) (Figure 7). Cross-linking of CD4 + T-cells by GP120 causes the induction of enhanced susceptibility to Fas-mediated killing. In the previously activated cells, GP120 cross-linking results in apoptosis (possibly mediated by IFN, TNF or both), downregulation of Bcl-2 expression and activation of caspase 3. GP120 can also induce apoptosis in uninfected CD4 + T-cells. Circulating immune complexes and replication-incompetent viruses that contain GP120 can also induce death in a similar manner [327]. GP120 can activate the Bax-dependent apoptotic mitochondrial pathway, which involves Puma, Bax, Bak, Cytochrome C, etc. The interaction of GP120 with CXCR4 can also cause MMP (mitochondrial membrane permeabilization) through pertussis toxin-sensitive G protein (Gia) P38 MPAK pathway and/or through a Ca^++^ dependent mechanism [329].

Tat is an inducer of apoptosis in infected cells potentially by Fas-dependent mechanism, superoxide dismutase inhibition or activation of cyclin-dependent kinases. The ability of Tat to induce cell death has been demonstrated in vitro for neurons and CD4 + T-cell lines. Tat is readily secreted by infected cells, and cellular and humoral immunity to Tat may have protective effects against HIV progression [326]. Nef is essential for viral pathogenicity and HIV-encoded Nef has been suggested as a potential mediator of apoptosis. This proposal is supported by the fact that human infections with naturally occurring Nef deletion mutants lead to less rapid CD4 + T-cell depletion [330].

HIV-1 encodes a small gene known as Vpr (viral protein regulatory) whose product is a 96-amino acid protein. HIV-encoded Vpr also has the ability to induce apoptosis through transfection and exogenous treatment. A proposed mechanism includes the induction of G2/M cell cycle arrest and a direct effect on mitochondrial permeability. When cells become infected with HIV-1, two deleterious effects result from the expression of the Vpr gene. One effect is to manipulate the cell cycle by blocking the cells in G2 phase (the phase of the cell cycle immediately preceding mitosis). Thus, cells infected with HIV-1 cease to proliferate. The second effect is the induction of apoptotic cell death. Vpr induces apoptosis via the intrinsic pathway. This pathway is characterized by cytochrome C release, and caspase 9 activations and is triggered in the absence of death receptor ligation [331]. A recent study characterized the dynamics of the morphological changes during G2 arrest by Vpr and apoptosis. Murakami and colleagues conducted time-lapse imaging of HeLa cells containing the fluorescent ubiquitination-based cell cycle indicator 2. They highlighted the effects of Vpr on G2 arrest and that subsequent apoptosis was reversible [332].

HIV-encoded protease is a cytotoxic protein that leads to apoptosis in human and bacterial cells after transfection. HIV protease correlates with the presence of apoptosis in vitro and in vivo. Findings suggest that HIV protease may also play a role in the death of HIV-infected T cells. However, there is no evidence that HIV protease can induce apoptosis in uninfected cells [326].

## HIV and UPR

HIV-1 Tat is a major culprit for HIV/neuro-AIDS. One of the consistent hallmarks of HIV/neuro-AIDS is reactive astrocytes or astrocytosis, characterized by the increased cytoplasmic accumulation of intermediate filament glial fibrillary acidic protein (GFAP). HIV-1 Tat induces GFAP expression in astrocytes and is responsible for astrocyte-mediated Tat neurotoxicity. Aggregation of GFAP induces UPR and endoplasmic reticulum stress. Interestingly, ER stress activation has only recently been detected in HIV-induced neurodegeneration. GFAP expression in the presence of Tat expression activated all UPR/ER pathways including PERK, IRE-1, ATF6 and OASIS [333].

HIV infection is capable of inducing BiP (chaperone immunoglobulin heavy chain binding protein) both in vivo and in vitro, potentially resulting in an environment that favors continued protein folding, which is a significant indication of UPR activation [334]. HIV infection was also shown to be an important factor contributing to higher levels of P-eIF2α in cells from an HIV patient, which leads to translational inhibition inside the cell [334]. HIV can also induce higher levels of IRE-1 phosphorylation, a hallmark of IRE-1 activation. One of the most important downstream effects of IRE-1 activation is the splicing of XBP1 mRNA, which in this condition will be translated as an important transcription factor that induces the expression of ER stress-response genes [334]. The interaction of HIV with the host apoptosis pathways in T-helper cells is illustrated in Figure 7 and a summary of these pathways and HIV infection are summarized in Table 7.

**Table 7.** Summary of HIV infection and autophagy, apoptosis and UPR pathways.

HIV proteins	Autophagy	Apoptosis	UPR
ENV (GP120-GP160)	Inhibition of autophagy in DCs, by activation of mTOR, silencing of LC3 and ATG5	Fas mediated killing, BCl down regulation which activates Bax dependant Mitochondrial pathway	GP120 increases the XBP-1 splicing, increases caspase 3 and caspase 9, increase in BiP and CHOP expression
Tat	Inhibition autophagy by interaction with LAMP2A→ stop autophagosome and lysosome fusion in CD4 + T-cells In glial cells inhibits autophagy by interaction with BAG3	Fas mediated killing, superoxide dismutase inhibition, cyclin dependant kinase activation	Accumulation of GFAP and induction of UPR by all 3 pathways; PERK, IRE1, ATF6 and OASIS. Tat increases the expression of ATF4 for the end of viral latency
Vpr		G2/M arrest → leads to apoptosis with increased BAX (mitochondrial protein) activation→ apoptosis via intrinsic pathway	
Nef	Interaction with IRGM ≤ assembly of ULK1/BECLIN-1/ATG6≤ autophagy induction	Apoptosis induction	
HIV protease		Induction of apoptosis by cleavage of host proteins	

## Concluding remarks

Cellular responses to stress are regulated via UPR and autophagy which is regulated by ER chaperone and autophagy regulatory proteins. Both UPR and autophagy are tightly involved in regulation of apoptosis in different cell models and can drive the cells to apoptotic cell death in chronic stress conditions or situations in which stress response mechanisms are not able to rescue the cells. Viruses induce host cellular stress response (UPR, and autophagy) and also are able to hijack autophagy and UPR machinery to promote host infection and replication. However, activation of autophagy and UPR via virus infection can induce chronic and persistent activation of cellular stress responses, which can be involved in the activation/modulation of the apoptotic pathway. Therefore, any tools (CRISPR, small molecule inhibitors/inducers) which can modulate autophagy and UPR could be beneficial approaches to target virus replicative cycles via apoptotic cell death, or by changing the cellular cytokine profile. Currently, many groups are focused on developing new therapeutic approaches to mitigate viral infection by targeting the autophagy and UPR pathways. Our team has recently showed that of autophagy pathway inhibition can successfully affect symptoms of viral infection in a hepatitis c drug irresponsive population [335]. Therefore, there could be a positive window to develop a new cocktail to target autophagy pathway for regulation of viral infection. UPR inhibitors have been successfully used in the animal model and some preclinical trial against different diseases. Recently, it has been showed that MKC8866 (inhibitor of IRE1) can affect secretome [336] therefore it may have some application in the regulation of immune response and cytokine profile of infected cells during viral infection. Overall, continued work in the next few years will better illuminate the roles of these pathways in the regulation of host responses to viral infection.
